# Collagen fiber regulation in human pediatric aortic valve development and disease

**DOI:** 10.1038/s41598-021-89164-w

**Published:** 2021-05-07

**Authors:** Cassandra L. Clift, Yan Ru Su, David Bichell, Heather C. Jensen Smith, Jennifer R. Bethard, Kim Norris-Caneda, Susana Comte-Walters, Lauren E. Ball, M. A. Hollingsworth, Anand S. Mehta, Richard R. Drake, Peggi M. Angel

**Affiliations:** 1 Department of Cell and Molecular Pharmacology, MUSC Proteomics Center, Bruker-MUSC Clinical Glycomics Center of Excellence, Medical University of South Carolina, 173 Ashley Ave, BSB358, Charleston, SC 29425 USA; 2 Division of Pediatric Cardiac Surgery, Vanderbilt University Medical Center, Nashville, TN USA; 3 Division of Cardiovascular Medicine, Department of Medicine, Vanderbilt University Medical Center, Nashville, TN USA; 4 Eppley Institute for Cancer Research and Allied Diseases, University of Nebraska Medical Center, Omaha, NE USA

**Keywords:** Congenital heart defects, Valvular disease, Peptides, Proteins, Proteomics, Protein-protein interaction networks, Mass spectrometry, Proteomic analysis, Cardiovascular biology, Congenital heart defects, Valvular disease

## Abstract

Congenital aortic valve stenosis (CAVS) affects up to 10% of the world population without medical therapies to treat the disease. New molecular targets are continually being sought that can halt CAVS progression. Collagen deregulation is a hallmark of CAVS yet remains mostly undefined. Here, histological studies were paired with high resolution accurate mass (HRAM) collagen-targeting proteomics to investigate collagen fiber production with collagen regulation associated with human AV development and pediatric end-stage CAVS (pCAVS). Histological studies identified collagen fiber realignment and unique regions of high-density collagen in pCAVS. Proteomic analysis reported specific collagen peptides are modified by hydroxylated prolines (HYP), a post-translational modification critical to stabilizing the collagen triple helix. Quantitative data analysis reported significant regulation of collagen HYP sites across patient categories. Non-collagen type ECM proteins identified (26 of the 44 total proteins) have direct interactions in collagen synthesis, regulation, or modification. Network analysis identified BAMBI (BMP and Activin Membrane Bound Inhibitor) as a potential upstream regulator of the collagen interactome. This is the first study to detail the collagen types and HYP modifications associated with human AV development and pCAVS. We anticipate that this study will inform new therapeutic avenues that inhibit valvular degradation in pCAVS and engineered options for valve replacement.

## Introduction

Congenital aortic valve stenosis (CAVS) accounts for 10% of all congenital heart defect cases, affecting up to 6 of every 1000 newborns^1^. There are two distinct subsets of this disease: (1) pediatric end-stage, characterized by a rapid engorgement of the structure with extracellular matrix (ECM), and (2) the more frequently studied adult end-stage, characterized by fibrocalcific lesions^2^. These defects result in a narrowing of the aortic opening, obstructing aortic outflow and leading to left ventricular hypertrophy and heart failure. Despite the clinical significance, there are no available therapeutics. The patient is managed by “watchful waiting” until deteriorating cardiac hemodynamics indicates a need for surgical intervention, either through valve replacement or heart transplantation^3^. Bioengineered aortic valve replacements are especially limited for pediatric patients due to their inability to grow with the patient, lifelong requirements of anti-clotting agents, and serial surgeries to replace valves during somatic growth^4^. There is an ongoing need for identifying therapeutic targets, particularly for the pediatric population.

Collagens form the underlying scaffolding of valvular structure that influences valvular function and consequently cardiac function^5–12^. Collagen is developmentally regulated and is required for appropriate heart function during somatic growth^12–14^. The normal human aortic valve has an ECM trilayer structure defined by a high density collagenous fibrosa facing blood outflow, an interior spongiosa composed of primarily glycosaminoglycans, and the elastin-rich ventricularis^7,12^. In the mature human valve, a parallel alignment of collagen with valvular endothelium is critical for normal function^7,13^, highlighting collagen organization within the trilayer compartments as fundamental to valvular function. Pediatric CAVS produces excessive collagen deposition that mixes with other ECM proteins, creating a disorganized ECM structure, altering valvular function and leading to cardiac failure^5,15,16^. Although collagen deregulation is a hallmark of adult and pediatric valvular stenosis^5,17,18^ and a potential prognostic and therapeutic target^19,20^, very little is known about the complexities of collagen fiber regulation in the human valve.

In the current study, we test the hypothesis that pCAVS disease mechanisms also include dysregulation of collagen fiber arrangements with an altered post-translational regulation. A total of 20 human AV tissue specimens with clinical data on AV function were evaluated. Histopathological stains paired with microscopy methods were used to define collagen fiber changes and collagen variation within the valvular structure. A collagen-targeting^21–24^ high-resolution accurate mass (HRAM) proteomics approach was used to measure collagen type composition and PTM regulation within the collagen structure. Protein–protein interaction analyses were done to determine possible upstream regulators relevant to collagen deregulation observed in pCAVS. Upstream regulators were validated via immunohistochemistry and RT-PCR. Results of this study indicate that pCAVS shows fundamental differences in collagen fiber organization, collagen type regulation, and regulation of proline hydroxylation of collagens. These studies are the first to report specific collagen regulation due to pCAVS, and establish a foundation for new translational and post-translational collagen studies in fibrotic cardiovascular disease.

## Results

### Clinical and morphological characteristics of study cohort

Collagen from human aortic valve samples was studied across three patient categories: normal, pediatric end-stage congenital aortic valve stenosis (pCAVS), and aortic valve insufficiency (AVI) (Table 1). Age-matched pediatric samples ranged from Neonates (2 weeks) to Adolescent (18 years), with an average patient age of 6.75 ± 6.06 years. Gender was predominantly male (15, 57.7%). The race was primarily Caucasian (16, 61.5%). For each patient, serial tissue sections were evaluated for histopathological distribution of collagen by multiple staining methods and collagen fiber measurements. Translational and post-translational regulation of collagen was investigated by collagen targeted proteomics^25^ using HRAM proteomics on regions approximately 100 µm in thickness adjacent to histological sections. In summary, this cohort represents a suitable pathological variation towards probing ECM distribution in human aortic valve development and pediatric end-stage disease.

**Table 1:** Descriptive statistics of patient characteristics for all pediatric aortic valve (AV) tissue samples featured in the study.

SKU	Age (years)	Pediatric age group	Gender	Race/ethnicity	BSA (m^2^)	Leaflet morphology	Aortic valve function
**Normal**							
DB017	0.5	Infant	M	–	0.38	Trileaflet	Normal
DB051	0.66	Infant	M	C	0.34	Trileaflet	Normal
NDRI	2	Young child	M	L	0.63	Trileaflet	Not recorded
DB85	9	Child	F	C	0.95	Trileaflet	Normal
DB27	17	Adolescent	M	C	2.2	Trileaflet	Normal
Normal Averages	7.17 ± 7.5		75%M 25%F	75%C 25%L	1.03		
**pCAVS**							
DB106	0.04	Neonate	M	C	0.21	Bicuspid	Severe stenosis with AVI
DB43	0.15	Infant	M	C	0.24	Trileaflet	Moderate stenosis with AVI*
DB40	0.75	Infant	M	C	0.37	Bicuspid	Moderate stenosis
DB105	5	Young child	M	C	0.72	Bicuspid	Moderate stenosis with AVI
DB119	5	Young child	F	–	–	Bicuspid	Moderate stenosis with AVI
DB113	5	Young child	–	–	–	–	Trivial stenosis; severe AVI
DB18	6	Child	F	C	0.7	Bicuspid	Moderate stenosis with AVI
DB3	11	Child	F	C	1.5	Bicuspid	Moderate stenosis with AVI
DB117	12	Adolescent	M	–		Bicuspid	Moderate stenosis with AVI
DB41	14	Adolescent	M	C	1.6	Bicuspid	Moderate stenosis with AVI
DB16	17	Adolescent	M	C	2.1	Bicuspid	Moderate stenosis with AVI
pCAVS Averages	6.90 ± 5.8		70%M 30%F	100%C	0.93		
**AVI**							
DB114	0.17	Infant	F	–	–	Trileaflet	Trivial AVI
DB007	0.58	Infant	–	–	–	Trileaflet	Mild nAVI
DB108	10	Child	F	C	1.3	Trileaflet	Thickened with AVI
DB83	10	Child	F	AA	1.4	Trileaflet	Trivial AVI
DB032	16	Adolescent	–	–	–	Trileaflet	Mild nAVI
AVI Averages	7.35 ± 6.8		–	–	1.35		

Normal AVs are from cardiac transplant patients where normal AV function is define by 2D echocardiogram. 2D Echocardiogram mean gradients define pre-operative AV function where a normal gradient is defined as < 5 mmHg, mild stenosis as 5–25 mmHg, moderate stenosis as 25–50 mmHg, and severe stenosis as > 50 mmHg. BSA: body surface area; C: Caucasian; L: Latino; AA: African American; MD: Myxomatous Degeneration. DB43 was included in pCAVS due to stenosis, but main diagnosis was Hypertrophic Cardiomyopathy. Pediatric Age Groups are defined by National Institute of Child and Human Development: Neonate (0–30d), Infant (1 mo–2yrs), young Child (2–6 yrs), Child (6–12 yrs), Adolescent (12–18 yrs). Experiment information can be seen in Supplemental Table S5.

### Pediatric CAVS valves trend to be collagen dominant at a younger age of end-stage

**Figure 1:**
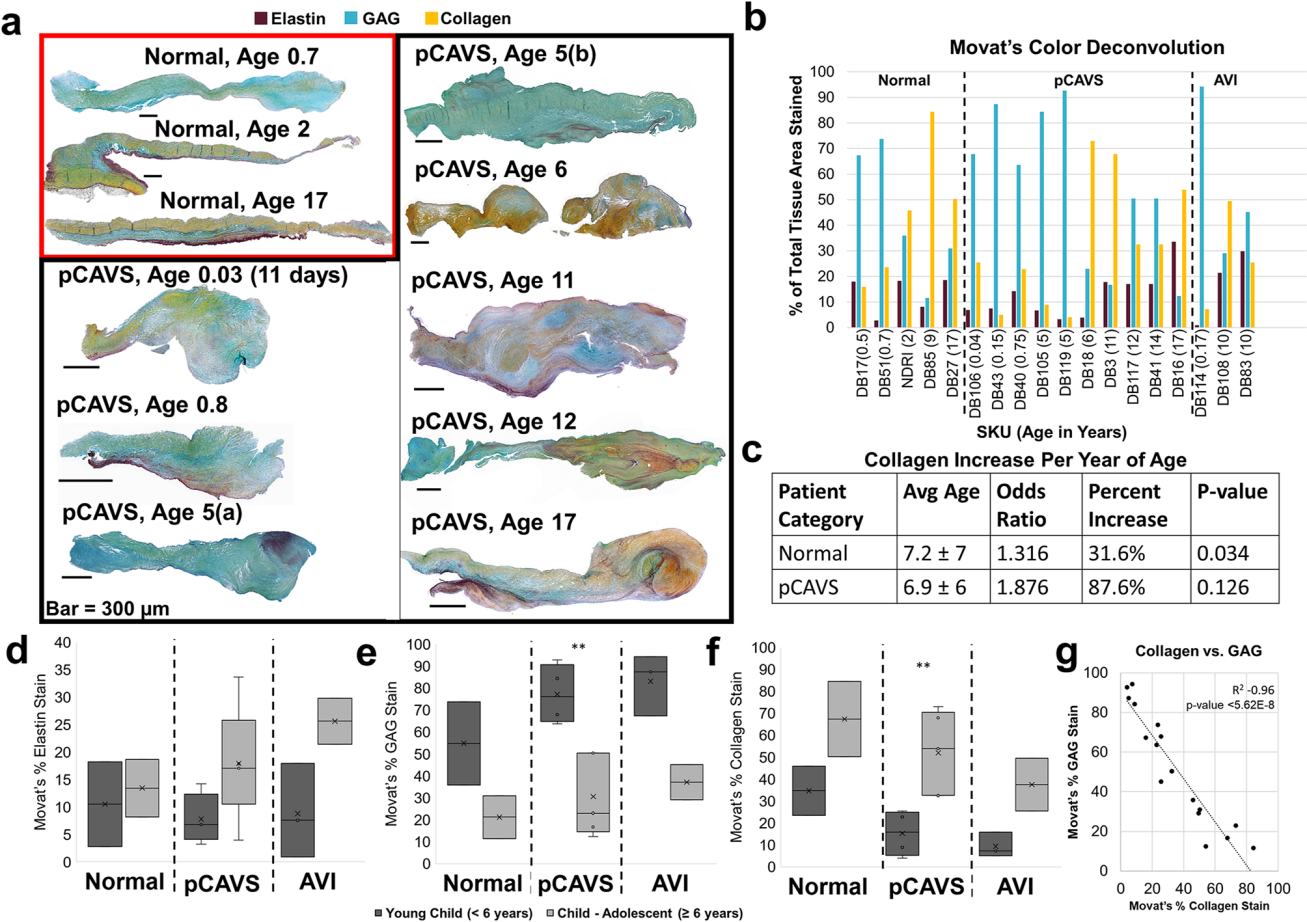
Histology analysis identifies ECM mixing and collagen dominance in pCAVS valves at a young end-stage. (**A**) Examples Movat’s Pentachrome staining between normal and age matched pCAVS samples. Blue stain corresponds to glycosaminoglycan (GAG) regions, dark purple to elastin, and yellow to collagen. Normal valves show traditional trilayer structure, while pCAVS valves show lack of trilayer and areas of ECM mixing. (**B**) Quantification of Movat’s Staining via color deconvolution analysis. Blue = GAG, purple = Elastin, yellow = collagen. Shown as a percent of total Movat’s stain area. (**C**) Multinomial logistical regression analysis of Movat’s Pentachrome Color Deconvolution data shows pCAVS valves are collagen dominant at a younger age than normal patients. Variables: ECM dominant category (GAG, elastin, or collagen); Patient Category (normal or pCAVS); Age (0–18 years). (**D**–**F**) Quantification of Elastin, GAG, and Collagen (respectively) Movat’s Pentachrome color deconvolution results (Fig. 1B) comparing two pediatric age groups: young child (Newborn to < 6 years of age) and Child-Adolescent (6–18 years of age). **p-value 0.008, MWU test. pCAVS GAG decrease is concomitant with collagen increase across pediatric age categories (**G**) Scatterplot of all data points across Normal, pCAVS, and AVI samples for collagen vs. GAG stain percent of total area of Movat’s stain. Bar: 500 μm. Spearman’s correlation (R^2^) − 0.96 (* p*-value < 5.62E–8). Normal n = 5; pCAVS n = 10; AVI n = 3.

Color deconvolution of Movat’s Pentachrome on the valve cohort was performed to determine the dominant ECM type in each patient category (Fig. 1A, B, Supplementary Fig. S1). Movat’s Pentachrome stain showed an ECM trilayer in the normal valves with thickened, disorganized ECM due to pCAVS, consistent with previous literature reports on CAVS^5,26^. We stratified the pediatric tissues by neonatal to early childhood (0-including 5 years of age; rapid growth and development) and middle childhood to early adolescence (age 6–18 years of age). While differences in elastin can be seen from patient to patient, elastin content was not statistically significantly different between patient categories (Fig. 1B, D). Pediatric patients in all categories showed decreased glycosaminoglycan (GAG) staining over time relative to patient age (Fig. 1E). GAG decrease was significant within the pCAVS category compared between two patient age groups: young children (77.2 ± 14%; pediatric age < 6 years, n = 6) vs children to adolescents (30.6 ± 19%; pediatric age > 6 years, n = 5) (*p*-value 0.008) (Fig. 1E, F). Collagen content increased concomitant with decreasing GAG content over all patient groups (Spearman’s correlation − 0.96; *p*-value < 5.62E–8) (Fig. 1G). Interestingly, while collagen content progressively increased with age for each patient category, the likelihood of a pCAVS valve having a collagen dominant phenotype occurred at a younger age of end-stage. Histopathology evaluation revealed that the rate of collagen deposition increased by 56% in pCAVS compared to normal AV development. When accounting for age by multinomial logistical regression, analysis showed that the probability of a normal valve being in the collagen dominant ECM category increased by 32% (*p*-value 0.034, Fig. 1C) for every year of age. In contrast, pCAVS valves showed a trend to having a collagen dominant phenotype which increased by 88% (*p*-value 0.126, Fig. 1C) for every year of age. Collagen significantly increased in between pediatric patient age groups (34.7 ± 15.7% age < 6 years; 67.4 ± 24.1% age ≥ 6 years, *p*-value 0.0079) (Fig. 1F). These results show that the ECM disorganization of pCAVS involves a progressive collagen dominant phenotype concomitant with GAG decreases.

### Pediatric CAVS demonstrate high deposition of immature collagen compared to individual age-matched controls

**Figure 2:**
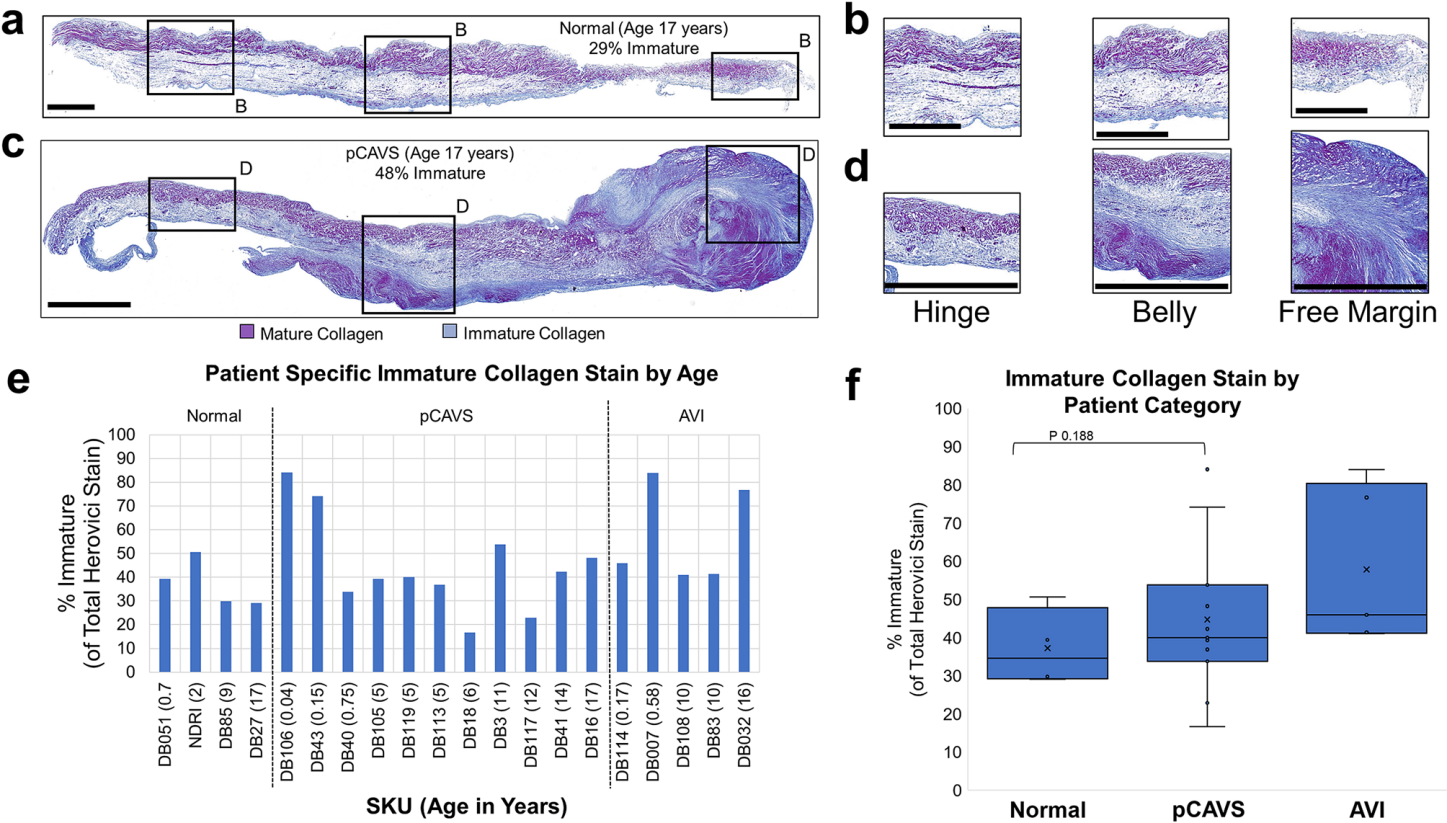
Pediatric CAVS demonstrate high deposition of immature collagen compared to age-matched controls. (**A**,**B**) Herovici staining of SKU DB27, a 17-year-old normal sample with regions of Hinge, Belly, and Free Margin 10 × zoom shown (**B**). Blue = immature. Purple-mature. Normal immature collagen production is localized to the ventricularis, while pCAVS immature production becomes increasing dysregulated towards the free margin. (**C**,**D**) Herovici staining of SKU DB16, an age-matched pCAVS sample with regions of Hinge, Belly, and Free Margin 10 × zoom shown in (**D**). (**E**) Patient specific breakdown of Herovici color deconvolution analysis. pCAVS patients show increased immature collagen production compared to age matched controls. (**F**) Boxplot showing quantification of Herovici color deconvolution, showing larger heterogeneity amongst pCAVS patients. Scale Bar: 300 μm. Normal n = 4; pCAVS n = 11; AVI n = 5.

Herovici stain was used to assess valvular content of pre-collagen or immature collagen (blue) and mature collagen (purple) (Fig. 2, Supplementary Fig. S2; all valves studied)^27^. Immature collagen deposition varied by location on the valve structure and by pathology, suggestive of a link to mechanics of valve function. In trilayer regions close to the hinge region, normal valves and pCAVS showed immature collagen deposition in the spongiosa (Fig. 2A–D). Within the belly of the leaflet, immature collagen deposition aligned with regions of ventricularis delamination. However, pCAVS showed sites of pathological mixtures of immature and mature collagen. For example, in SKU DB16, immature collagen appears mixed with mature collagen along the ventricularis closer to the hinge. The free edge of this valve contains significant mixtures of both immature and mature collagen, suggestive of a dynamic production of collagen in this region. By Herovici stain, all valves showed new production of collagen (Fig. 2F). Averaged across ages at end-stage, there was no significant difference between normal and pCAVS levels of immature collagen. However, comparison of same age-matched patients showed increases in immature collagen (ex. 19% increase in SKU DB16 vs DB27) (Fig. 2E). Overall, the data show that both normal and pCAVS continually produce immature collagen with pCAVS showing pathological mixes of both immature and mature collagen.

### Pediatric end-stage CAVS valves have greater numbers of collagen fibers with localized regions of collagen density

**Figure 3:**
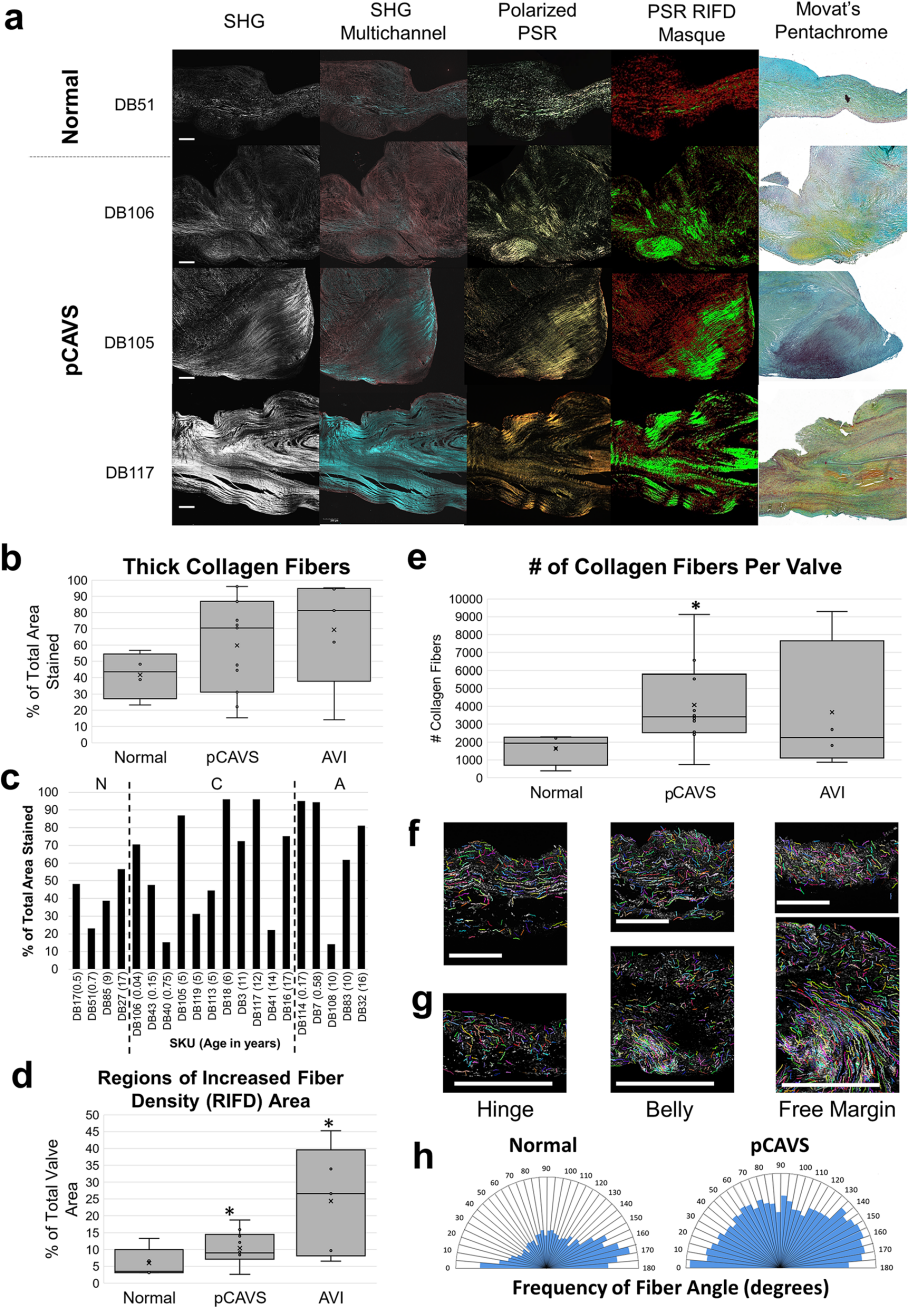
Pediatric end-stage CAVS valves have a greater number of collagen fibers with localized regions of collagen density. (**A**) Collagen fiber visualization of select pCAVS and normal valves. From left to right: Second Harmonic Generation (SHG); SHG multichannel; Polarized picrosirius red (PSR); PSR Regions on Increased Fiber Density (RIFD) Masque; Movat’s Pentachrome. Movat’s Pentachrome: blue = GAGs, yellow = collagen, and purple = elastin. Scale Bar = 200 μm. (**B**) Boxplot quantification of picrosirius red color deconvolution analysis, showing percent thick stain over total PSR stain. (**C**) Patient-specific data of PSR color deconvolution analysis, showing percent thick stain over total PSR stain. pCAVS and AVI patients show larger heterogeneity across age groups compared to normal. (**D**) Boxplot quantification of collagen RIFD areas normalized to total valve area. *p-value 0.024, MWU test. pCAVS and AVI patients have significantly more regions of high collagen fiber density compared to normal patients. (**E**) Boxplot showing quantification of total number of collagen fibers as measured by CT-FIRE analysis. *p-value 0.014, MWU test. pCAVS valves have significantly more collagen fibers than normal valves. (**F**,**G**) Hinge, Belly, and Free Margin (respectively) 10 × zooms of PSR-polarized light signal overlayed with CT-FIRE mask of collagen fibers for normal (F; SKU DB27) and pCAVS (G; SKU DB16) (Supplmentary Figure S3). Normal valves show stratified collagen fibers throughout the leaflet, while pCAVS valves show densely deposited fibers increasing in destratification from hinge to free margin. (H) Polar histogram showing frequency distribution of collagen fiber angles from 0–180°, across all normal valve samples (left) and pCAVS (right). pCAVS valves have a large distribution of fiber angels indicating destratification, while normal valve collagen fiber angles stratify to 0° and 180° relative to the endothelium. Normal n = 4; pCAVS n = 11; AVI n = 5. CT-FIRE V2.0 Beta was used (https://eliceirilab.org/software/ctfire/).

Collagen fiber measurements were done by Picrosirus Red (PSR) staining to characterize collagen fiber regulation (thickness, density, and alignment) (Fig. 3; Supplementary Fig. S3). Interestingly, all pCAVS valves showed localized areas of high-density collagen, confirmed by second harmonic generation (SHG) microscopy (Fig. 3A, Supplementary Fig. S3). Collagen fiber thickness was not significantly different between normal valves compared to pCAVS or AVI. However, larger interquartile ranges were observed in pCAVS (37–81% thick fibers) compared to normal valves (34–50% thick fibers), suggesting that pCAVS have increased heterogeneity of fiber thickness (Fig. 3B, C). Compared to normal valves, both pCAVS and AVI valves demonstrated a significant increase in regionalized collagen density normalized by area (*p*-value 0.024 and 0.019) (Fig. 3A, D). Localized areas of high-density collagen fibers did not appear to be specific to valvular anatomy and occurred at different locations within the leaflet. The amount of collagen fibers in pCAVS valves was significantly higher when compared to normal valves (2.5-fold more,* p*-value 0.014; normalized to cross-sectional area; Figure 3E). Picrosirius Red staining, in combination with CT-FIRE software, was used to determine the collagen fiber angle alignment relative to endothelium. Collagen fiber angles in normal valve structures were primarily between 150–180° relative to endothelium (SKU DB27, Fig. 3F, H), following previous observations in normal valve growth^7,13,17^. In comparison, the pCAVS valves showed a larger distribution of fiber angles (over 0–180° relative to the endothelium) along the length of the valve, suggestive of collagen fiber misalignment compared to normal controls (Fig. 3G, H). To summarize, the data shows that pCAVS valves are characterized by localization of high-density collagens and higher number of collagen fibers compared to normal valves.

### Proteomic analysis indicates collagen structure is post-translationally regulated in pCAVS

**Figure 4:**
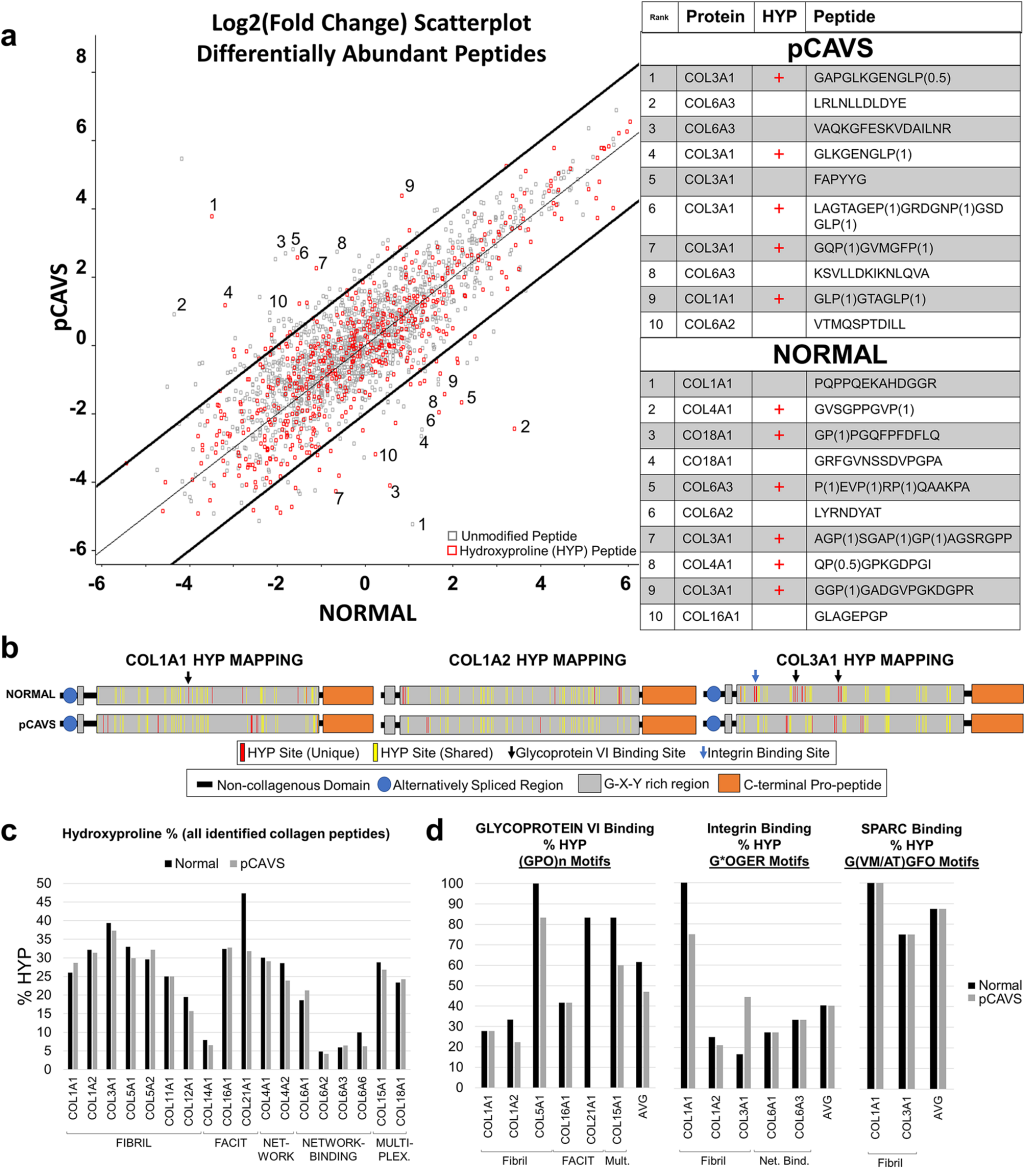
Pediatric CAVS valves have reduced HYP, including in collagen-ECM binding sites. (**A**) A log2(fold change) scatterplot of pCAVS versus Normal peptides. Red squares are hydroxyproline containing peptides, gray squares are unmodified peptides. Bold lines indicate X + 2 and X − 2. Top 10 collagen peptides are annotated with corresponding sequence and protein shown (**A**, right). P(X) where X is between 0–1, indicated HYP probability at that site. Significantly regulated collagen subtypes differ between pCAVS and normal, with non-fibril types (multiplexin, FACIT) differentially regulated in normal. (**B**) Representative hydroxyproline site mapping for collagen 1A1, collagen 1A2, and collagen 3A1, shown for Normal and pCAVS patients (DB27 and DB16, respectively). Normal sample contained more HYP sites than age matched controls. Unique HYP sites in Normal (top, red) are found in glycoprotein vi and integrin binding motifs (black and blue arrows, respectively). HYP is required in these motifs for binding. (**C**) Quantification of hydroxyproline state of proteins in all peptides identified in normal (black) or pCAVS (gray). (**D**) Hydroxyproline percent in peptides containing glycoprotein vi, integrin, or SPARC binding motifs. Loss of HYP may indicate lack of collagen-ECM binding. FACIT: Fibril-associated collagen with interrupted triple helices; Net.: Network Collagen; Net. Bind: Network Binding Collagen; Mult.: Multiplexin (Multiple triple-helix domains with interruptions). Normal n = 4, pCAVS n = 11. MaxQuant and Perseus v.1.6.3.3. was used for this analysis (https://maxquant.net/maxquant/).

A collagen- and ECM protein-targeted proteomic analysis approach was next applied to the valve tissue cohort in order to understand how collagen fiber regulation corresponds with collagen protein structure regulation. The proteomics analysis workflow is summarized in Supplementary Figure S4 and described in Materials and Methods. A focus of the analysis was testing for hydroxylated proline (HYP) residues, a collagen post-translational modification essential to triple helical stability and collagen organization^28,29^. From a total of 2985 ECM peptides identified, 126 peptides were differentially expressed between normal and pCAVS by a minimum of twofold change (Fig. 4, Supplementary Tables S1, S2). The majority of altered peptides were from collagen type proteins (87/126; 69%) and these included site-specific modifications of hydroxylated proline (Fig. 4A). Hydroxyproline site mapping from age-matched normal and pCAVS valves suggested a strong potential for HYP site variation within the triple helical regions on primary fibril type collagens COL1A1, COL1A2 and COL3A1 (Fig. 4B). Site occupancy analytics showed 41 HYP sites in COL1A1 were shared between normal and pCAVS valves; the normal valve showed 5 unique sites of HYP modification while the pCAVS valve showed 8 sites of HYP modification that were not detected in normal valves (Fig. 4B). Consistent with data across the abundantly changed peptides, normal valve derived COL1A2 and COL3A1 contained more HYP sites than the age-matched pCAVS valve. In COL1A2, 41 sites were shared, with 9 unique HYP sites in normal valve and only 3 unique HYP sites in the age matched CAVS sample. However, for COL3A1, the normal AV (SKU DB27) showed an additional 16 unique HYP sites, with only 7 unique HYP sites identified in the pCAVS valve (SKU DB16; Fig. 4B). Among patient groups, the overall percent change in HYP content showed more subtle variation (Fig. 4C), yet it is likely that regulation at key HYP sites has greater effects on collagen structure organization^28,30^. specific HYP binding site motifs were evaluated for pCAVS related changes. These included motifs for glycoprotein VI (collagen-induced platelet adhesion and activation), integrin (cell-surface receptor for collagen)^31^ and SPARC (cell invasion) (Fig. 4D)^30^. Qualitatively, fibril type collagens COL1A2 and COL5A1 showed reduced HYP in glycoprotein VI binding sites. Similarly, COL1A1 demonstrated potential reduction in HYP content for integrin binding sites (Fig. 4D). COL21A1, which was recently found to be transcriptomically increased in cardiomyopathy remodeling^32^, showed large decreases in pCAVS HYP content; in pCAVS, HYPs in known binding motifs of glycoprotein VI were undetected in COL21A1 (Fig. 4D). The analysis of collagen structure variation by peptide analytics suggests that collagen HYP site regulation may contribute to human aortic valve status.

### Valve status is characterized by a differential ECM proteome with primary activities of collagen interaction

**Figure 5:**
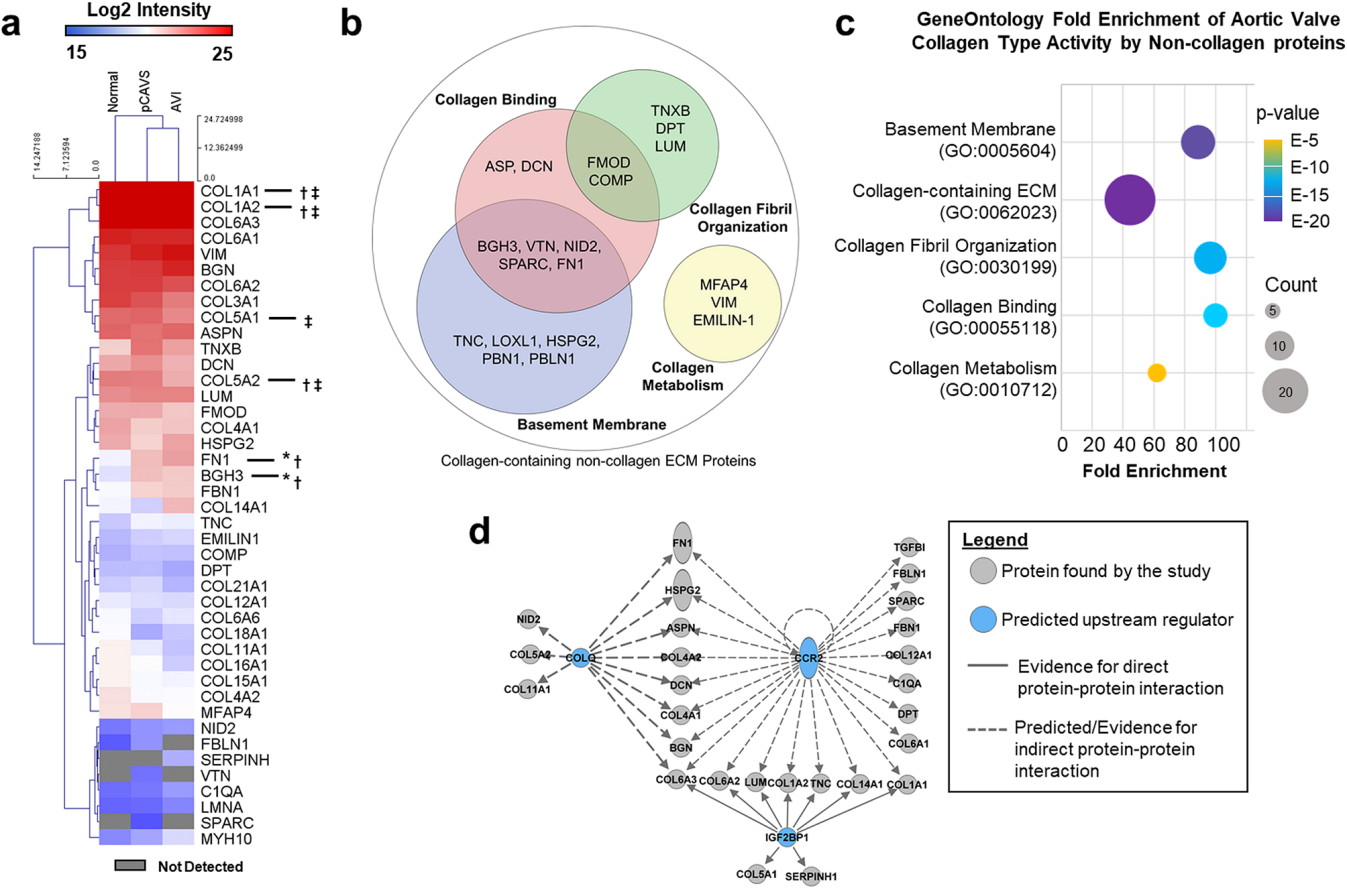
Extracellular matrix proteomics implicates collagen binding and collagen organization pathways. (**A**) Aortic valve extracellular matrix proteins identified by the collagen-targeting proteomics. A total of 18 collagen proteins were identified with COL1A1, COL1A2 and COL6A3 being the most abundant collagens. Protein level quantification, which includes all peptides both unmodified and modified, revealed significant differences based on valve diagnoses in COL1A1, COL1A2, COL5A1, BGH3, FN1, and COL5A2. *Mann–Whitney U adj. *p*-value < 0.05 normal AV compared to pCAVS, †Mann–Whitney U adj. *p*-value < 0.05 normal AV compared to AVI, ‡Mann–Whitney U adj. *p*-value < 0.05 AVI compared to pCAVS. (**B**) Gene Ontology Functional classification of non-collagen type proteins identified in the proteomics dataset. (**C**) Gene Ontology Fold Enrichment analysis of collagen type function by non-collagenous proteins identified by proteomics, Fisher’s exact*p*-value ≤ 1.84E–5, false discover rate ≤ 6.66E–3. Proteins associated with collagen binding had the highest degree of fold enrichment, followed by collagen fibril organization. (**D**) Upstream analysis of the ECM proteome by Ingenuity Pathways Analysis to identify potential regulators that explain directional changes in aortic valve ECM proteins. Primary regulators affecting the proteome were C–C Motif Chemokine Receptor 2 (CCR2; Fisher’s Exact*p*-value 1.13E–39), Collagen Like Tail Subunit Of Asymmetric Acetylcholinesterase (COLQ; Fisher’s Exact*p*-value 9.67E-19), (Insulin Like Growth Factor 2 MRNA Binding Protein 1 (IGF2BP1, Fisher’s Exact*p*-value 1.05E–18). Normal n = 4, pCAVS n = 11.

Protein level analysis was performed on the proteomic data (Fig. 5, Supplementary Table S3) to further evaluate how non-collagen ECM proteins contribute to collagen regulation. Proteomic analysis identified a total of 44 ECM proteins, including 18 collagen type proteins, many of which have not been previously associated with the human aortic valve (Fig. 5A). When measuring total protein abundance, most collagens showed very little change between normal and pCAVS, suggesting that regulation at the level of post-translational modifications may play a larger role in AV collagen organization. However, there were multiple ECM proteins associated with collagen regulation detected as differentially regulated. The abundance of the proteins fibronectin (FN1) and Transforming Growth Factor Beta Induced protein (BGH3; cell-collagen adhesions^33^) were significantly increased in pCAVS compared to normal AV (2.5-fold, MWU*p*-value 0.04; 3.4-fold, MWU* p*-value 0.02, respectively). In AVI, COL1A1 and FN1 increased compared to normal AV tissue (1.6-fold, MWU* p*-value 0.02; 4.6-fold, MWU* p*-value 0.03). Additionally, the proteomics identified SPARC and VTN proteins only in the pCAVS proteomes. Gene ontology analyses identified that 26 of the 29 non-collagen proteins were associated with collagen-containing extracellular matrix (Fig. 5B, C, Supplementary Table S4). Furthermore, very specific groups of proteins were involved in collagen binding, collagen fibril organization and collagen metabolism (Fig. 5B, C).

To understand potential regulators of the dataset responsible for directional expression changes in pCAVS compared to normal, protein interaction networks were interrogated (Fig. 5D). While some regulators, such as TGFβ1, are known to be associated with ECM remodeling and deposition^34^, three novel upstream regulators of the collagen proteome were identified that could account for change in expression patterns. This included C–C Motif Chemokine Receptor 2 (CCR2; Fisher’s Exact *p*-value 1.13E–39), Collagen Like Tail Subunit Of Asymmetric Acetylcholinesterase (COLQ; Fisher’s Exact* p*-value 9.67E-19), and (Insulin Like Growth Factor 2 MRNA Binding Protein 1 (IGF2BP1, Fisher’s Exact*p*-value 1.05E–18). Both CCR2 and COLQ1 were implicated as inhibited in pCAVS based on directional changes^35,36^. On the whole, proteomics identified new collagen types in the human valve and demonstrated that changes in pCAVS structure may identify target regulators of the collagen proteome.

### BAMBI as a potential novel ECM regulator of pediatric congenital aortic valve stenosis

**Figure 6:**
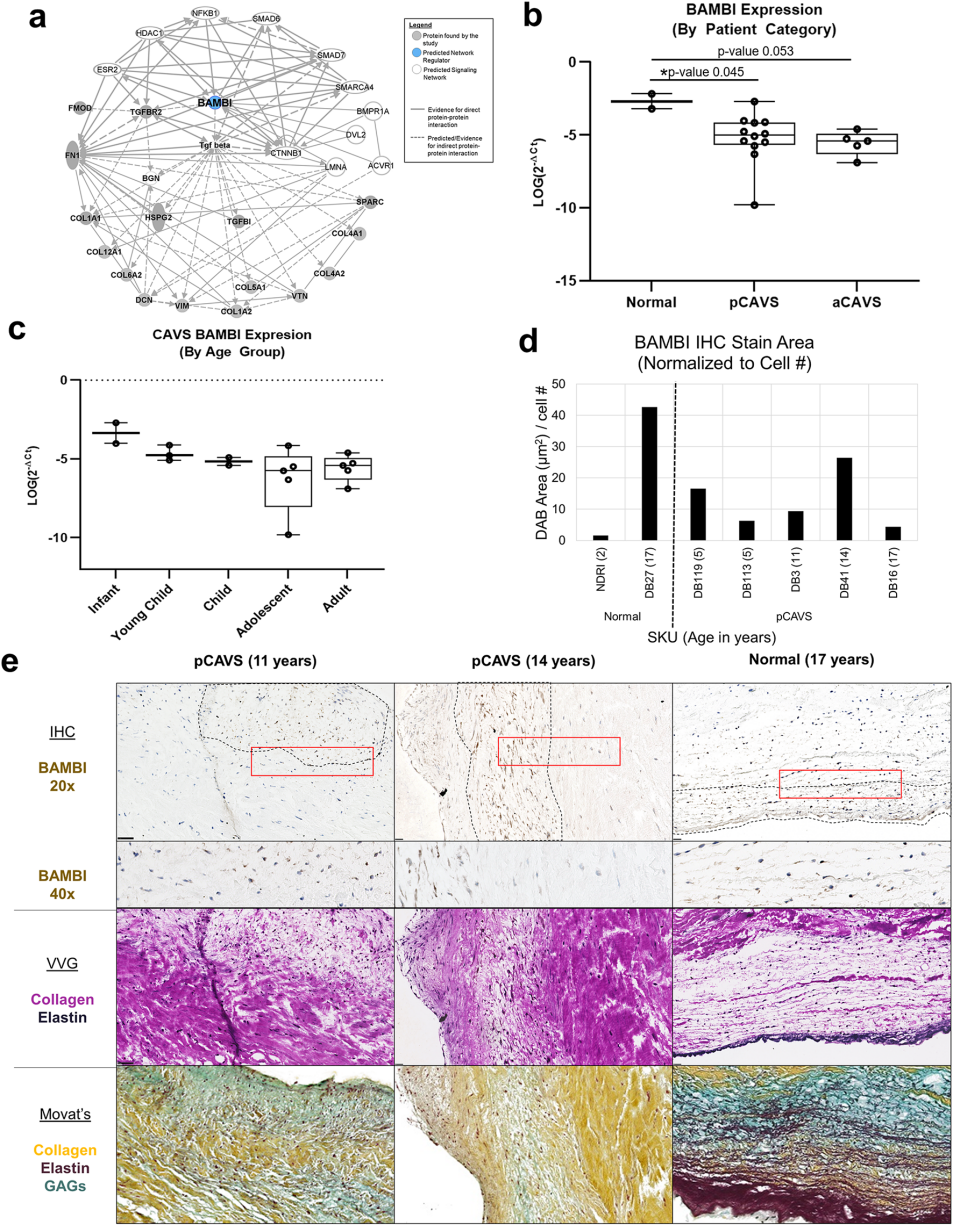
Proteomics based protein–protein interaction networks identify BAMBI as a potential master regulator of the collagen interactome in pCAVS. (**A**) Protein–Protein Interaction network showing relationship BAMBI (Supplemental Figure S5) relative to the ECM proteome and key valvular development proteins (Supplemental Table S6). (**B**) Quantification of qRT-PCR expression data on BAMBI (normalized to GAPDH), between patient categories. One-way ANOVA shows significant differential expression between patient categories (*p-value 0.013), with significant reduction in pCAVS (MWU *p-value 0.045) and trending reduction in aCAVS (MWU *p*-value 0.053) compared to normal pediatric patients. (Normal n = 2; pCAVS n = 12; aCAVS n = 5) **C.** Quantification of qRT-PCR expression data on BAMBI (normalized to GAPDH) of CAVS patients, compared between pediatric age categories (Infant n = 2; Young Child n = 3; Child n = 2; Adolescent n = 5; Adult n = 6). A suggestive trending decrease of BAMBI expression as a function of pediatric age categories is seen (ANOVA *p*-value 0.096) (Table 1; Supplemental Table S7). (**D**) DAB stain area quantification of BAMBI IHC of select valves in the cohort. (**E**) Representative immunohistochemistry staining of BAMBI in a selection of pCAVS and normal valves 20× (top) and 40 × zoom are shown. BAMBI positive cells appear highly localized, shown in dotted lines. Verhoeff van Geison (VVG) staining on the same valve section post-IHC study is shown (middle). Movat’s Pentachrome staining of a serial valve section (bottom). Positive BAMBI staining in pCAVS corresponds heavily to areas low in collagen staining (VVG) and high in GAG staining (Movat’s), while normal valve shows positive staining in collagen and elastin rich regions.

To identify master regulators that could be driving previously identified upstream regulators of the AV collagen proteome, causal network regulation analysis was performed. This analysis expands the scope of the upstream regulator analysis from Fig. 5 by integrating literature-observed cause-effect relationships (Fig. 6, Supplementary Figure S5). One such master regulator putatively identified was BMP and Activin Membrane-Bound Inhibitor homolog (BAMBI;* p*-value of overlap 1.16E–18) (Supplementary Fig. S5), previously identified as both developmentally regulated^37^ and a negative regulator of TGFβ1^38,39^. Further studies have found that BAMBI may be biomechanically sensitive, and functions to protect murine heart from pressure overload through restraint of TGFβ signaling^40^. In this study, BAMBI was identified as having a high *p*-value of overlap, which unlike z-score-sorted regulators, is identified independently of the dataset’s regulation weight or direction^41^. This preliminary identification of BAMBI was confirmed to have both direct and indirect interaction with proteins implicated in valve disease and development, such as ACVR1, DVL2, and SMARCA4 (Fig. 6A; Supplementary Table S6) Confirmational transcription-level analysis was performed on a cohort of fresh-frozen pediatric and adult aortic valve samples (Supplementary Table S7, Fig. 6b, c, Supplemental Figure S5). Adult CAVS samples (aCAVS) used in this study showed no calcification by clinical annotation, with the except of SKU SB176. BAMBI expression was found to be significantly differentially regulated between patient categories of normal pediatric, pCAVS, and aCAVS (ANOVA *p*-value 0.013), with significant reduction in pCAVS (*p*-value 0.045) and trending reduction in aCAVS (*p*-value 0.053) compared to normal pediatric patients (Fig. 6b). Amongst CAVS patients, there is a suggestive trending decrease of BAMBI expression as a function of pediatric age categories (ANOVA *p*-value 0.096) (Fig. 6c).

IHC staining of BAMBI was performed to visualize translational expression relative to ECM staining in age-matched samples (young child age 2–5; adolescent age 12–18) (Fig. 6D,E; Supplementary Figs. S6, S7). BAMBI was not abundantly detected in young normal valves (SKU NDRI, age 2) nor, in contrast, older adolescent pCAVS valves (SKU DB16, age 17) (Supplementary Fig. S6, S7). In the adolescent normal valve sample, BAMBI was observed in ventricularis endothelium and in regions of elastin delamination, as visualized by Verhoeff van Gieson (VVG) staining of the same tissue section (Fig. 6E, right). BAMBI was also detected in spatially localized regions of mixed collagen-GAG, seen in Movat’s Pentachrome staining (Fig. 6e). In pCAVS, only certain cells were BAMBI-positive and tended to be within the fibrosa-spongiosa mixed interstitium of GAG associated regions low in primary collagen (Fig. 6E, left and center columns). These data suggest that future studies on BAMBI may show its contribution to regional collagen deposition within the valve structure.

## Discussion

Collagen forms the fundamental framework of the aortic valve starting from embryonic developmental stages onward; disease-driven changes deform the framework and ultimately lead to heart dysfunction^10,14,42–44^. In pCAVS, rapid and excessive production of ECM components increases leaflet thickness by up to eightfold, leading to pediatric heart failure^5,16,45,46^. Genomic studies, primarily characterized from adult end-stage phenotypes, show complex genetics with dissimilar patterns of inheritance, genetically heterogenous phenotypes, and multiple chromosomal regions that impact the ECM network directly or indirectly^45,47–49^. Gender may also play a role—while bicuspid aortic valve is three times as likely to be found in males, females have more collagen deposition and less calcification^50,51^. Surgical replacement of valves is an option in adults, but in children, valve replacements do not grow with the patient, leading to heart dysfunction and failure^4,52^. Directly or indirectly targeting collagen production in pediatric end-stage CAVS may be a viable therapy inhibiting the disease, but very little is known about translational and post-translational regulation collagen and ECM components that form the working structure of the maturing aortic valve. The goal of this study was to identify the dynamic collagen regulation that occurs in pCAVS, including collagen sub-type regulation, collagen fiber alignment, and regulation of hydroxylated proline residues associated with triple helical stability.

The ECM trilayer structure was lost in pCAVS and the rate of collagen deposition trended to increasing by up to 56% per year compared to normal AV development (Fig. 1). This coincides with previous reports that pCAVS involves overproduction of collagen to result in thickened valves^5,10,16^. Supporting this, immature collagen fibers were detected by staining in all ages of pCAVS localized to regions of mixed collagen and elastin, particularly valves that showed hypertrophic commissures. Significantly, the pCAVS valves showed that collagen fiber alignment with the endothelium is lost and resulted in a random fiber angle relative to endothelium that localized with stains for immature collagen fibers. An additional finding was that unique, pathologically confined regions of increased collagen fiber density were observed in all pCAVS structures (Fig. 3). This contrasted with measurements on normal valves in the study, where collagen fiber angles were parallel to the endothelium with the fibrosa staining for mature collagen fibers. Similar findings in normal human aortic growth have been reported, with emergence of collagen fiber alignment parallel to endothelium within maturing fibrosa^7^. Previous work in normal human aortic and pulmonary valve has shown that fibrosa collagen is circumferentially oriented, and that uniformly distributed, densely packed collagen fibers emerge with age^13^. Age-related increases in collagen density in pCAVS were attributed to an imbalance of normal collagen synthesis and remodeling required to maintain appropriate collagen stretch dependent on local hemodynamics^13^. We also observed that for valves with aortic valve insufficiency, higher but more variable levels of thick collagen fiber production were observed compared to either normal or pCAVS valves, supporting that valvular function is a balance of collagen production and collagen degradation^10–12^ with dependence on hemodynamics. Additional but unknown effects on collagen production and alignment may result from the cell type and density composition within the tissue microenvironment, in addition to the differential effects of hemodynamics throughout the pCAVS structure. Alterations in collagen fiber alignment are an important component of the pCAVS end-stage and are a useful target in mechanistic studies on the disease.

Collagen PTMs are numerous and include non-enzymatic glycation, lysine and proline crosslinking, N- and O-linked glycosylation, and tyrosine sulfation^28,53,54^. Here, we focused on hydroxylation of proline (HYP) to understand collagen regulation in pCAVS. HYP is a primary collagen PTM that regulates triple helical stability and consequently has a large influence on collagen organization in tissue^28^. Regulation of HYP sites within the collagen structure alters fiber conformation and integrity and works to promote or protect the collagen structure from degradation^28,54–56^. HYP is regulated by co-factors of iron, oxygen, and ascorbic acid, with dependency on nutritional status and oxidative stress ^1,28,56–58^. Cell-specific and disease-specific regulation of prolyl hydroxylases P4HA1, P4HA2, P4HA3 drive HYP modification on collagen^58,59^. Cell recognition of very specific sites of hydroxylated prolines along the collagen fiber results in signaling changes through integrins and the tyrosine kinase receptors discoidin domain receptors (DDRs)^60–63^. These cell-fiber interactions influence epithelial mesenchymal transition, cell proliferation survival and metastatic expansion^61,62,64–66^. The current study identified up to 15% reduction of HYP content in pCAVS, dependent on collagen subtype. However, HYP changes appeared to be site specific. It is possible that changes in HYP sites may contribute to the rapid collagen degradation and remodeling of pCAVS^28^. Further, the sites of HYP loss in the pCAVS group aligned with regions of glycoprotein VI and integrin binding, which require the presence of HYP within the sequence. Integrins play a crucial role in cell-ECM interactions, with recent data suggesting a key role in dynamic connective tissue remodeling events during wound healing^67^. Glycoprotein VI is a platelet specific glycoprotein that has been shown to have affinity for collagen in the dimeric form^68^. It has been reported that adult patients with AVS have decreased platelet function and aggregation, however the role of HYP content has not been explored as a potential mechanism^69^. This study identifies that HYP regulation on primary fibrillar collagens may contribute to intrinsic and extrinsic mechanisms of pCAVS and merits focused studies.

Histological studies identified localized regions of dense collagen formation in common with pCAVS. Protein level studies gave insight into the collagen composition of the pCAVS deregulated structures. Collagen alpha-2(V) (COL5A2), was found to have significant increases pCAVS compared to non-stenotic AVI valves. Importantly, COL5A2 has been reported within heterotypic Collagen Type 1 fibrils and plays an essential role in regulating fibrillogenesis and the size of collagen fibrils^70^. The histopathological finding of high-density collagen fiber regions in pCAVS may be reflective of the increased fibrillogenesis. However, to implicate COL5A2 directly, spatially oriented proteomics would be required to understand localization and regulation of the highly density collagen sites compared to collagen type composition. With localized molecular biomarker identification, these regions of dense collagen fibers provide a potential mechanism of early-stage AV fibrosis molecular marker detection in vivo^71^. Amongst non-collagen type proteins, the enrichment of proteins associated with collagen binding, collagen fibril organization, and basement membrane was consistent with our histopathological findings of collagen fiber misalignment and HYP regulation in binding sites. Interestingly, collagen chaperone SPARC was found to be enriched in pCAVS compared to normal AV and AVI patients, independent of age (Fig. 5a). While never explored in healthy or disease AV, the overexpression of SPARC has been reported with under-expression of COL4A1 and other network collagens in myocardium. Increases in SPARC has been linked to endothelial dysfunction, possibly via its binding to VCAM-1 and altered integrin interactions downstream^72^. Its abundance in pCAVS may suggest endothelial dysfunction. Research of healthy and diseased endothelial cell signaling in AV is ongoing, however direct correlation between VEC or VIC cell activation and corresponding collagen regulation remains to be explored^16,73,74^.

The study identified BAMBI as a novel candidate that may be involved in valvular biology. BAMBI is a negative regulator of TGFβ1 signaling^38,39^ and positively modulates Wnt/β-Catenin pathways by increasing interactions with frizzled 5 and disheveled segment polarity protein 2 (DVL2)^37^. While never studied directly in AV disease or development, recent studies suggest that BAMBI is regulated in the myocardium to restrain hypertrophy and fibrosis^40^. In the current study, network analysis highlighted BAMBI as a predicted node amongst proteins known to be involved in valvular development and disease. Interestingly, the initial protein-level staining studies demonstrated that the human aortic valve has cell-specific and localized expression of BAMBI, particularly within the glycosaminoglycan-rich spongiosa. Furthermore, by transcriptional analysis of total valvular content, BAMBI expression appeared to decrease in both pediatric and adult CAVS. It is conceivable that decreases in BAMBI may play a role in the excess ECM that is a hallmark of pCAVS. However, the role of BAMBI in regulating valvular biology remains undefined.

The sample size of the cohort was limited. Pediatric tissues inherently are difficult to obtain with a past national average of only 40% of families consenting for tissue use and donation^75^. Actual figures in specific regions may be lower^76^. This limitation similarly effects pediatric human aortic valve interstitial cell studies, as these require fresh samples for immediate cell isolation^77,78^ as opposed to the resected valves that are available through tissue banks. Nevertheless, this is the largest collagen study to date on valve tissue with pediatric end-stage aortic valve stenosis. Future investigations to extend studies from this small clinically-relevant cohort will be critical to understanding how developmental timing of valvular interstitial cell signaling influences spatial distribution of collagen fiber production.

## Conclusions

This study is the first to identify a tissue associated collagen signature unique to pediatric end-stage aortic valve stenosis, which includes highly localized regions of dense and realigned collagen fibers within the valve structure. Additionally, this is the first quantitative and structural report of collagen hydroxyproline, or HYP, in valve development and disease. These data establish the groundwork for future studies examining the spatial regulation of collagen HYP within valvular structures and in relation to signaling factors driving collagen organization and deposition in pCAVS. We anticipate that this data will inform functional testing of cell-based mechanisms regulating collagen HYP, the generation of new mouse models of valve disease, and improvements in the development of collagen biomaterials used in in vitro valve disease models and bioengineered valve replacements.

## Methods

### Materials

Xylenes, 200 proof ethanol was from Fisher Scientific (Pittsburgh, PA, USA). Acetonitrile, Ammonium Bicarbonate, Calcium Chloride, Formic Acid, and Trizma Base were from Sigma-Aldrich (St. Louis, MO, USA). PNGase F Prime was purchased from N-Zyme Scientifics (Doylestown, PA). Collagenase type III (COLase3) (*C. histolyticum*) was purchased from Worthington Biochemical (Lakewood, NJ, USA).

### Tissue procurement

Aortic valve tissue samples were procured through the Vanderbilt Core Laboratory for Translational and Clinical Research and the National Disease Research Interchange. The aortic valve tissues were collected under the Vanderbilt Pediatric Congenital Heart Disease Biorepository, written informed consent was obtained, and the project was approved by the Vanderbilt Institutional Review Board (IRB) and the IRB at the Medical University of South Carolina. De-identified tissues were obtained during reparative or transplant surgeries and characterized by pre-operative function into three patient categories: normal tricuspid (normal), pediatric CAVS bicuspid (pCAVS), and aortic valve insufficiency tricuspid (AVI). Valvular function is from de-identified complete 2D, color and spectral Doppler, or M-mode echocardiogram reports acquired prior to valve resection, replacement or heart transplant. The pCAVS patients were identified as being stenotic, predominantly bicuspid, and having mild aortic valve insufficiency. The AVI group only has aortic valve insufficiency, but are not stenotic and are all tricuspid. Adult CAVS samples (aCAVS) used in the qRT-PCR study showed no calcification by clinical annotation, with the except of SKU SB176. A complete list of studies performed on each tissue are listed in Supplementary Tables S5 and S7. Pediatric ages used to stratify the population are based in National Institute of Child Health and Human Development (NICHD) pediatric terminology defining pediatric age groups in terms of clinical treatment, considering rapid somatic growth stages^79^.

### Histological staining

The 5-µm thick formalin-fixed, paraffin-embedded tissue sections were stained with Picrosirius Red (PSR; Polysciences, Inc., Warrington, PA, USA), Movat’s Pentachrome and Herovici (American Mastertech Scientific of StatLab, McKinney, TX, USA) following manufacturer’s protocols. Optical light whole tissue scans were done using a high-resolution slide scanner (Nanozoomer, Hamamatsu, Japan). Polarized light images of PSR were taking with a Spinning Disk CARV II microscopy (BD Biosciences, San Jose, CA, USA), and manually stitched together using Photoshop (Adobe, San Jose, CA, USA). Movat’s Pentachrome, PSR, and Herovici Color Deconvolution analysis was done using ImageJ^80^ adjusting for RGB hue threshold color (Movat: Elastin 0–19, 170–255; GAG 80–169; Collagen 20–79) (PSR: Thin 46–190; Thick 0.45, 191–255) (Herovici: Immature 0–167; Mature 168–255). Percent of each color threshold is represented as a percent of total area stained (RGB 0–255). RIFD (Region of Increased Collagen Fiber Density) analysis was done using ImageJ^80^. Briefly, polarized light images were converted to 8-bit and thresholded to include all signal. Images were processed with a mean filter of 1 μm and a look-up table of Red/Green was applied. Regions of increased collagen fiber density (RIFD) were quantified by thresholding to include the top 50% most dense signal (i.e., “green” component). Collagen fiber analysis (fiber length, width, angle) of PSR birefringent images were converted to 8-bit grayscale and analyzed using CT-FIRE^81^.

Second Harmonic Generation (SHG) microscopy was performed on the same tissue section used for PSR studies. SHG images were collected with a Spectra-Physics InSight X3 dual laser system and excited at 860 nm. SHG images and were collected at emission 432 nm with a 45 nm bandpass. SHG multichannel show overlay images collected at emission wavelengths: 432 nm (± 22.5 nm) (cyan; collagen SHG), 518 nm (± 22.5 nm) (green; collagen autofluorescence), 610 nm (± 35 nm) (red; PSR, elastin, and heme structures).

For immunohistochemistry, tissues were deparaffinized, antigen retrieved at pH 6 in sodium citate, then stained with HRP/DAB (ABC) Detection IHC Kit (Abcam, Cambridge, UK) according to manufacturer’s instructions with the following modifications: all incubation times were increased to 15 min. Primary antibody BAMBI (polyclonal) was diluted 1:100 (Millipore Sigma, St. Louis, MO, USA). IHC stained tissues were counterstained with Mayer’s hematoxylin (Electron Microscopy Sciences, Hatfield, PA, USA).

### Proteomic tissue preparation

Proteomic studies used tissue sections adjacent to histological studies (~ 150 μm distance). Tissues were 5-µm thick and mounted on standard microscope slides (Tissue Tack, Polysciences, Inc., Warrington, PA, USA). For proteomic experiments, slides were heated at 60 °C for 1 h then dewaxed as follows: xylenes (3 min, two times), 200 proof ethanol (1 min, two times) 95% ethanol (1 min), 70% ethanol (1 min), HPLC water (3 min, two times). Valve surface area was used to determine equivalent amount of tissue was scraped with a razor off the slide into centrifuge tubes. 10 mM Tris pH 9 was added to each tube to cover the tissue. Tissues were ultrasonicated at 50% energy (Fisherbrand 120 sonic dismembrator;Fisher Scientific, Pittsburgh, PA, USA) for 2 min each and incubated at 60 °C for two hours to antigen retrieve formalin-fixed crosslinks^82^. Samples were buffer exchanged into water and deglycosylated with 2 μg of PNGaseF PRIME (N-zyme Scientifics, Doylestown, PA) for two hours at 38 °C at 450 rpm using a thermomixer (Eppendorf, Hamburg, Germany). Supernatant was removed, and samples were buffer exchanged into 10 mmol/L ammonium bicarbonate, 3 mmol/L CaCl2, pH 7.25 and treated with 4 μg of Collagenase Type III (COLase3) overnight, shaking at 38 °C. An additional 4 μg of COLase3 was then added to each sample and incubated for 5 h at 38 °C with mixing at 450 rpm. COLase3 activity of 3 Units/mL was confirmed prior to digestion by a colorimetric activity kit (ab196999, Abcam, Cambridge, MA). Samples were centrifuged at 16,000 g for 5 min at 4 °C and supernatant collected for proteomic analysis. Samples were purified by C18 STAGE tip^83^ (Pierce Biotechnology, Waltham, MA) followed by a C18 ZipTip (Millipore Scientific, Burlington, MA) according to manufacturer’s protocol. Solutions were modified for STAGE tip using 90% acetonitrile, 5% formic acid (initialize and elute); 5% acetonitrile, 5% formic acid (equilibrate and wash). STAGE-tip eluate was dried down via speed vac and resuspended in 0.1% TFA in water. An additional C18 ZipTip according to manufacturer’s protocol (Millipore Scientific) and sample resuspended in mobile phase A (2% acetonitrile, 0.2% formic acid).

### Proteomics

Peptides were analyzed by data dependent acquisition on an Orbitrap Elite mass spectrometer equipped with a LC Packing U3000 nano-LC system (Thermo Scientific). Peptides were loaded onto a trap column and separated on a 75 μm × 30 cm classic pulled tip column (C18-Reprosil-AQ Pur RP 1.9 µm particles, Dr. Maisch, GmbH) at 60 °C. The gradient was from 5 to 40% solvent B over 180 min, where solvent A was 0.2% v/v formic acid in water and solvent B was 98% acetonitrile with 0.2% v/v formic acid. An FTMS survey scan was acquired over a mass range of 400–1700 m/z at a resolution of 60,000 with an automatic gain control (AGC) target value of 10^6^, followed by CID MS/MS of the top 10 most intense ions in the ion trap. Dynamic exclusion was enabled with a repeat count of 1, repeat duration of 30 s, and exclusion duration of 180 s. Data were searched using MaxQuant version 1.6.3.3^84^ against the human database (42,106 entries downloaded May 5, 2017) and a subset 1783 entries with keywords used (collagen, elastin, aggrecan, gelatin, osteonectin, perlecan, plasminogen, and fibronectin). Parameters included unspecified proteolytic enzyme, precursor mass tolerance of ± 20 ppm, and fragment mass tolerance ± 0.8 Da. Methionine oxidation, asparagine and glutamine deamidation were included as variable modifications. Proteins were identified with FDR ≤ 0.05 and at least two peptides. Hydroxylated proline modification site localization probabilities were obtained by re-searching MS/MS using a restricted database of identified proteins and allowing for dynamic modification proline oxidation. Perseus^85^ was used for protein and peptide level analyses.

### Protein–protein interaction network analysis

Protein identifications were uploaded to Ingenuity Pathways Analysis (IPA; Qiagen Inc., https://www.qiagenbioinformatics.com/products/ingenuity-pathway-analysis) to identify putative regulators of the protein dataset. Networks were calculated using predicted relationships between regulators by* p*-value ≤ 0.01; a *p*-value < 1.0 × 10^–12^ was used for inclusion in the network. Upstream Regulator Networks were calculated using predicted relationships between regulators (CCR2, COLQ, IGF2BP1); a *p*-value < 1.0 × 10–18 was used for inclusion of the upstream regulator network. Causal Network analysis was done to identify Master Regulators of the data (BAMBI) and a *p*-value < 1.0 × 10^–12^ of overlap was used for inclusion in the network.

### Proteomic data analysis and statistics

Perseus^85^ was used for protein and peptide level analyses. Hierarchical clustering was performed in Perseus on ANOVA significant peptide intensities via a permutation-based FDR of 0.05 and 250 randomizations. Clustering parameters: Euclidean distance, average linkage, and k-means pre-processing. Peptide level quantification was determined using peak intensities from peptides scoring ≥ 75 after median normalization and considered as significant with a minimum two-fold change. Relative changes in protein abundance based on normalized protein intensities were evaluated by ANOVA and Mann Whitney U test, correcting for multiple comparisons (IBM SPSS Statistics, version 25). A Type I error probability of ≤ 0.1 was used to evaluate the significance of the result. MWU-test was used in histological studies to calculate raw *p*-values. For this small clinical cohort,* p*-values of ≤ 0.1 are reported as trending to significance and *p*-values of ≤ 0.05 were used determine significant results.

GO enrichment was performed through Geneontology.org^86,87^. Genes were clustered to cellular component, biological process, or molecular function using Panther v14^88^ filtered by Fisher’s Exact (FE) *p*-values < 0.001 and false discovery rates (FDR) of < 0.005. Exported peak intensities are visualized as heatmaps after natural log transformation with MultiExperiment Viewer (http://www.tm4.org)^89^.

### Real time quantitative reverse transcription PCR

Aortic valve (AV) specimens were collected at the time of surgery or from an explanted heart. Samples were immediately flash-frozen and stored at − 80 °C till used for RNA extraction. Total RNA from AV tissues was isolated using RNeasy Kit (Qiagen, Germantown, MD), according to the manufacturer’s instructions. The BAMBI gene expression level was validated by quantitative RT–PCR (qRT-PCR) using the Taqman method, as previously described^90^. Briefly, cDNA was generated using 100 ng of total RNA and High Capacity cDNA Synthesize Kit (Catalog # 4,368,814 Thermo Fisher Scientific). Predesigned BAMBI (catalog # Hs03044164) and GAPDH (catalog # Hs02758991) primers were obtained from Thermo Fisher Scientific and the qRT-PCR were run in triplicates according to the manufacturer’s instructions. All Ct values were reported below 40, indicating acceptable PCR efficiency^91^.

## Supplementary Information

Supplementary Information.

## Data Availability

Any datasets generated during the study (not already in supplemental materials) are available on reasonable request. The mass spectrometry proteomics data have been deposited to the ProteomeXchange Consortium via the PRIDE^92^ partner repository with the dataset identifier PXD021990. During the peer review process, this data can be accessed via reviewer_pxd021990@ebi.ac.uk (Password: YA2RlOK6).

## Abbreviations

AVS: Aortic valve stenosis
pCAVS: Pediatric end-stage congenital aortic valve stenosis
AVI: Aortic valve insufficiency
ECM: Extracellular matrix
PTM: Post-translational modification
HYP: Hydroxyproline
HRAM: High resolution accurate mass
GAG: Glycosaminoglycan
