# Supplementary material for: Collagen fiber regulation in human pediatric aortic valve development and disease
Source: Sci Rep. 2021 May 7;11:9751. doi: 10.1038/s41598-021-89164-w (PMC8105334; doi:10.1038/s41598-021-89164-w)
Supplement: Supplementary file 1 — Supplementary Information. [file 41598_2021_89164_MOESM1_ESM.pdf]

Supplemental Materials

Collagen Fiber Regulation in Human Pediatric Aortic Valve Development and Disease

Cassandra L. Clift, BS[1], Yan Ru Su, MD, PhD[2], David Bichell, MD[3], Heather Jensen Smith[4], PhD, Jennifer Bethard,MS[1], Kim Norris-Caneda, MS[1], Susana Comte-Walters, MS[1], Lauren E. Ball, PhD[1], M.A. Hollingsworth, PhD[4], Anand Mehta, PhD[1], Richard R. Drake, PhD[1], Peggi M. Angel, PhD[1]*

1, Department of Cell and Molecular Pharmacology; MUSC Proteomics Center; Bruker-MUSC Clinical Glycomics Center of Excellence, Medical University of South Carolina, Charleston, SC

2, Division of Pediatric Cardiac Surgery, Vanderbilt University Medical Center, Nashville, Tennessee

3, Division of Cardiovascular Medicine, Department of Medicine, Vanderbilt University Medical Center, Nashville, Tennessee

4, Eppley Institute for Cancer Research & Allied Diseases, University of Nebraska Medical Center, Omaha, NE

*Corresponding Author: Peggi M. Angel, 173 Ashley Ave, BSB358, Charleston, SC 29425

Email: angelp@musc.edu Phone: (843) 792-8410 Fax: (843) 792-0481

Short title: Collagen types in human aortic valve

Total word count: 3946/4500 (not including Abstract, Methods, References, and Figure Legends)

Key words: aortic valve, congenital aortic valve stenosis, extracellular matrix, collagen, valve development, proteomics, collagen proline hydroxylation, collagen proteomics

Abbreviations: AVS, Aortic Valve Stenosis; pCAVS, pediatric end-stage congenital aortic valve stenosis; AVI, aortic valve insufficiency; ECM, extracellular matrix; PTM, post-translational modification; HYP, hydroxyproline; HRAM- high resolution accurate mass; GAG, glycosaminoglycan.

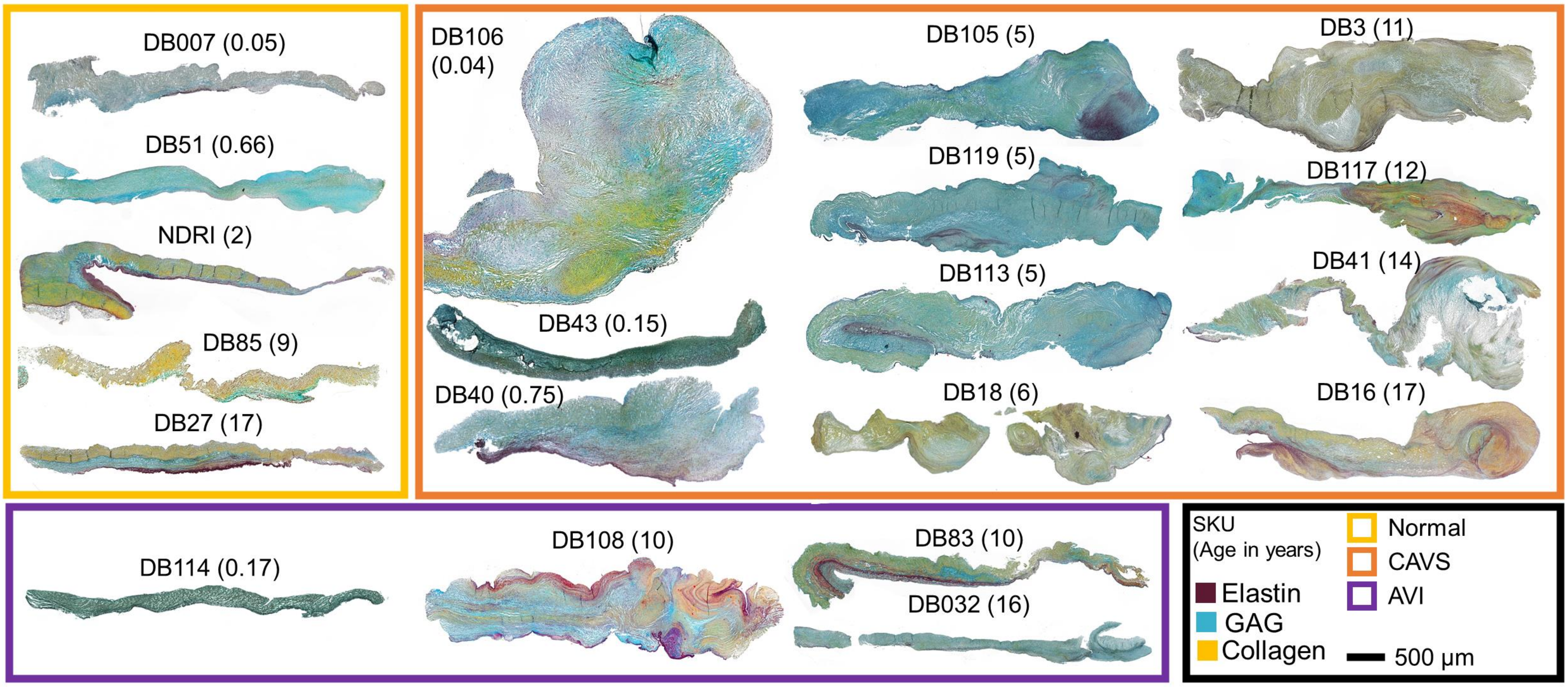

Supplemental Figure S1: Movat's Pentachrome images of all valves studied in Figure 1. DB113 and DB32 are included as representative images only, due to delayed acquisition and manufacturer's difference in sample preparation.

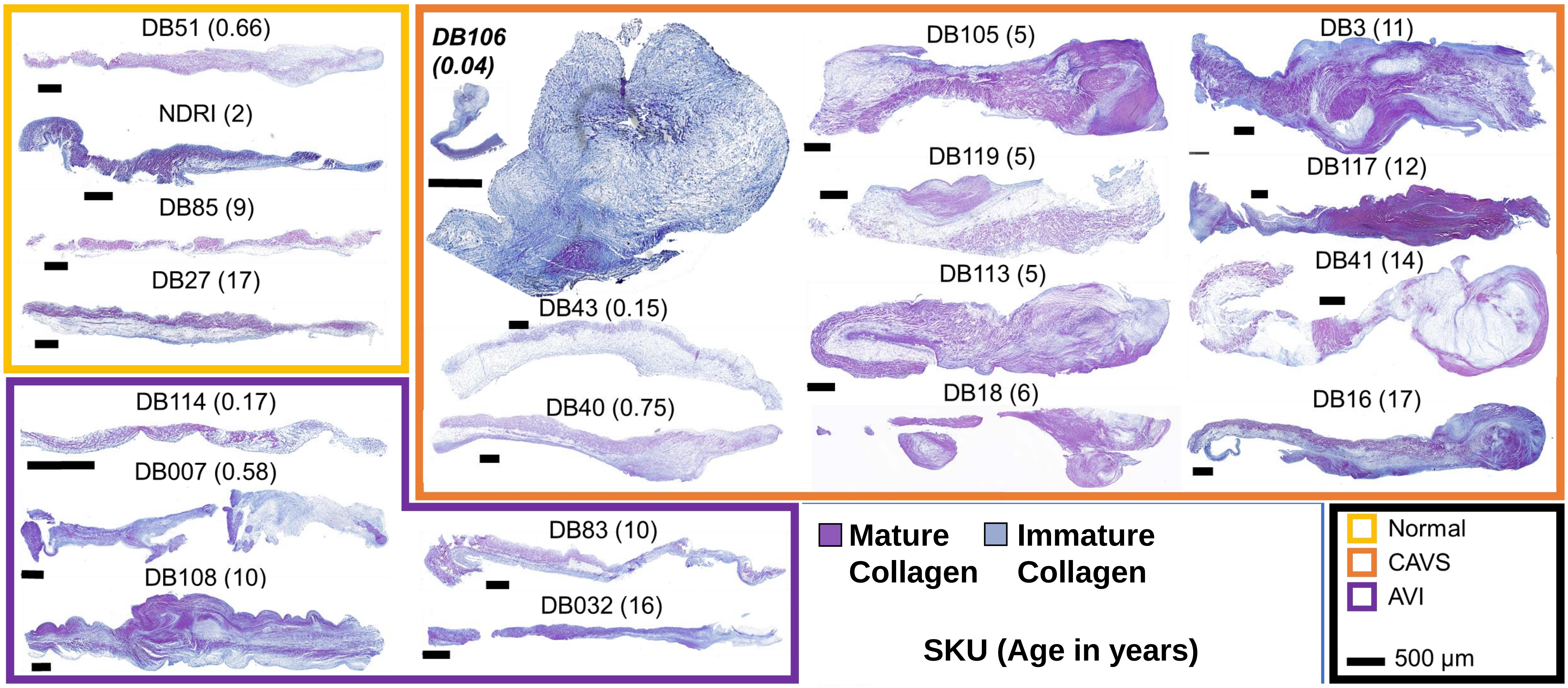

Supplemental Figure S2: Herovici images for all valves studied in Figure 2.

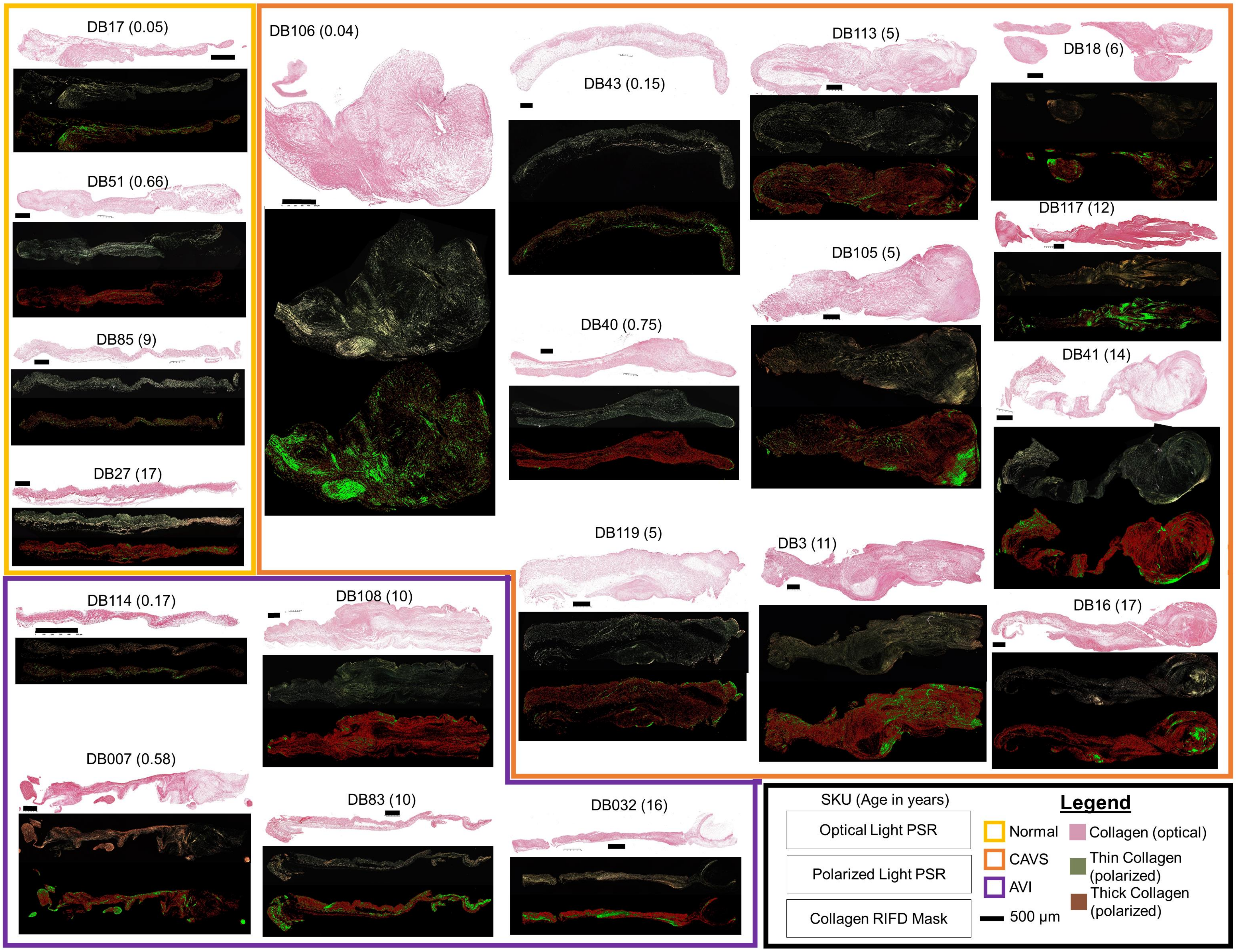

Supplemental Figure S3: Optical and Polarized Picrosirius Red (PSR) images of valves studied in Figure 3. RIFD (Regions of Increased Fiber Density) masks are shown.

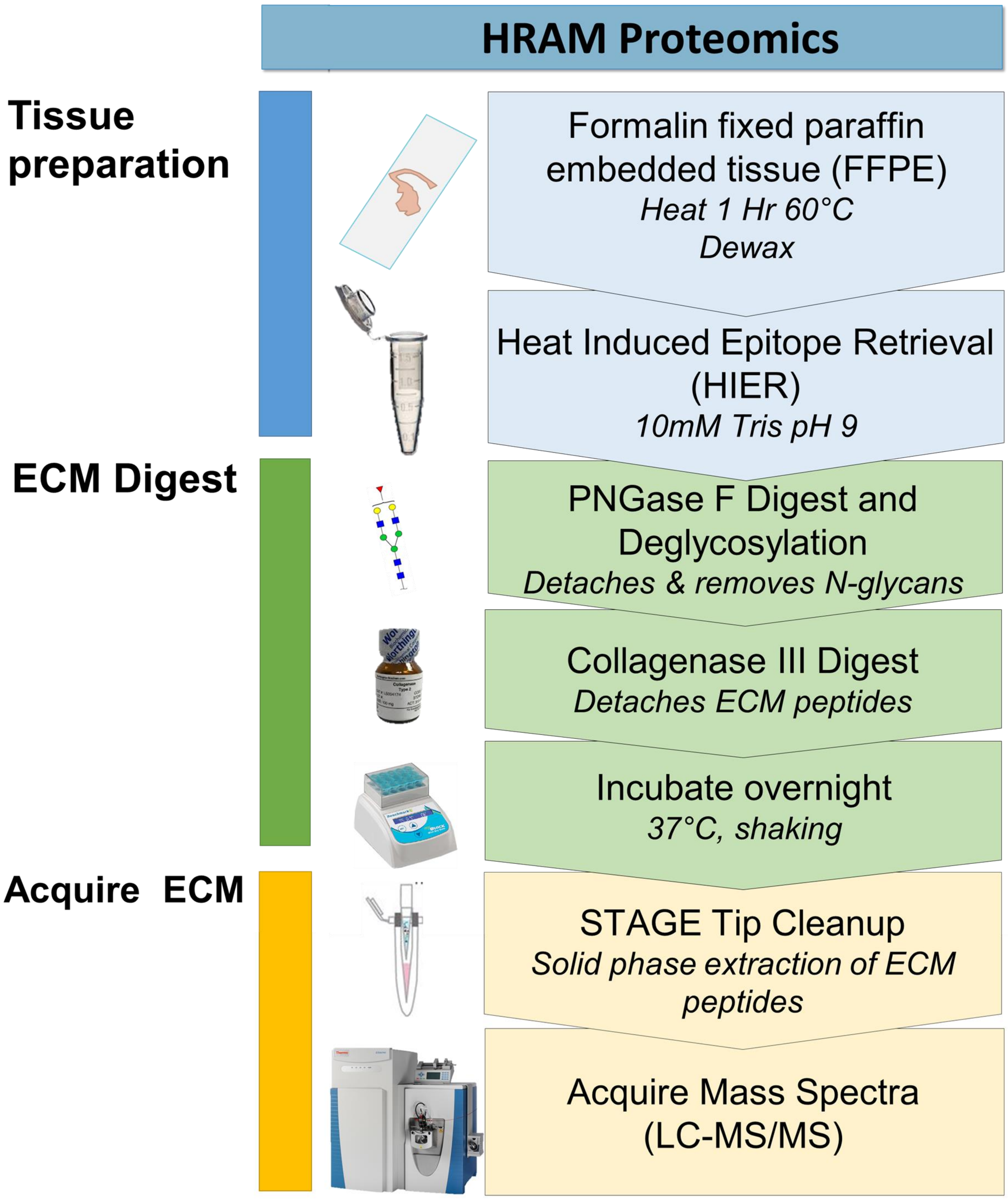

Supplemental Figure S4: Collagenase Type III high resolution accurate mass (HRAM) Proteomics workflow on formalin-fixed paraffin embedded tissue sections.

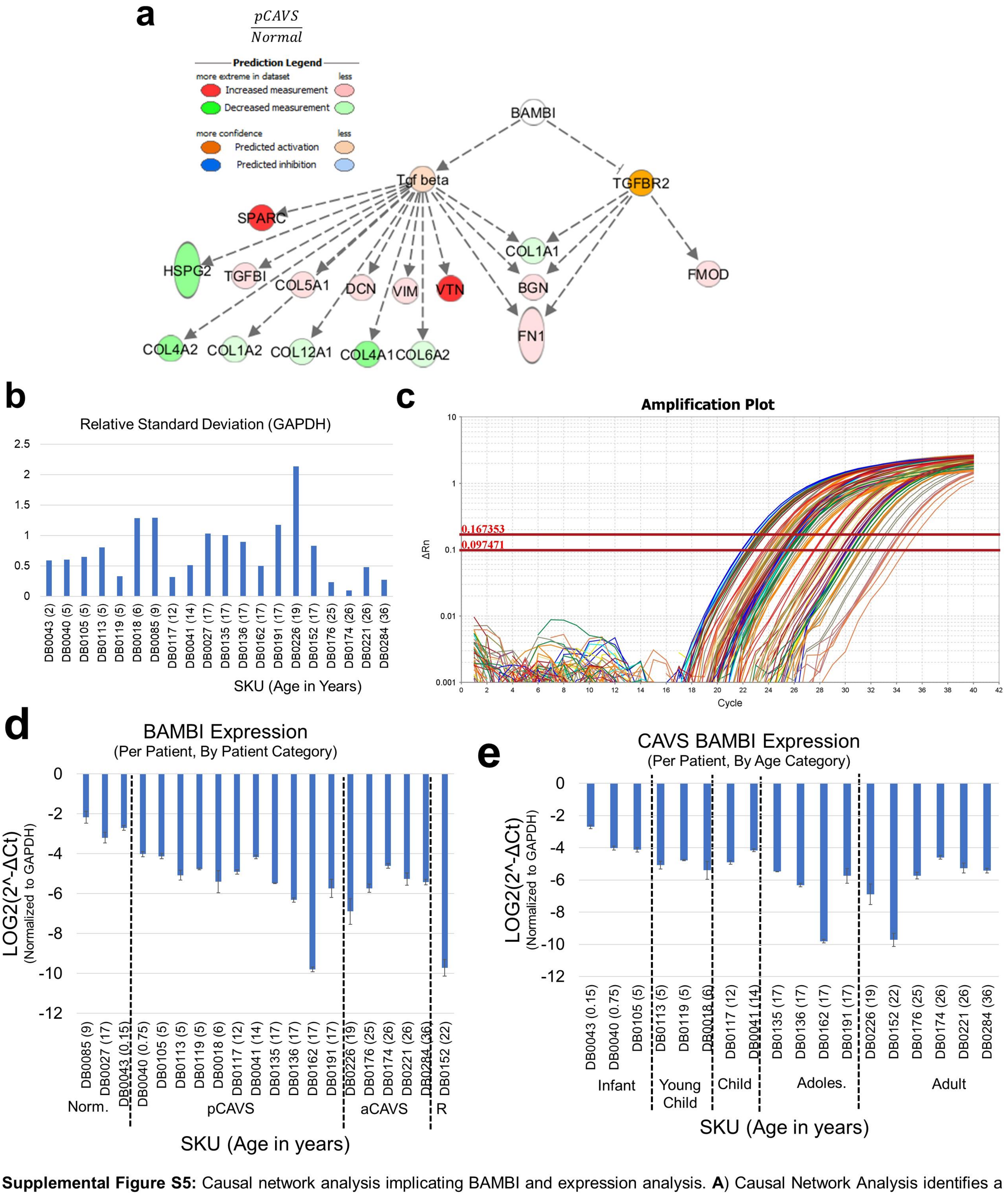

Supplemental Figure S5: Causal network analysis implicating BAMBI and expression analysis. **A**) Causal Network Analysis identifies a potential Master Regulator sorted by highest p-value overall and of overlap: BAMBI (BMP and activin membrane-bound inhibitor homolog, p-value 1.16E-18), based on trending expression patterns. **B**) Amplification plot of qRTPCR experiments in **Figure 6b-c; Supp Fig S5c-d**. **C**) Relative standard deviation (RSD) of GAPDH across all patient samples, sorted by age of patient. Average RSD is 0.75% ± 0.48%. **D**) Patient-by-patient expression (shown via LOG2($2^{-\Delta Ct}$), as normalized to GAPDH expression), organized by patient disease category. Norm: Normal; pCAVS: pediatric CAVS; aCAVS: adult CAVS; R: pulmonary valve by Ross procedure (**Fig. 6b**). **E)** Patient-by-patient expression (shown via LOG2($2^{-\Delta Ct}$), as normalized to GAPDH expression), of CAVS patients organized by pediatric age classification (**Fig 6c; Table 1; Supplemental Table S7**)

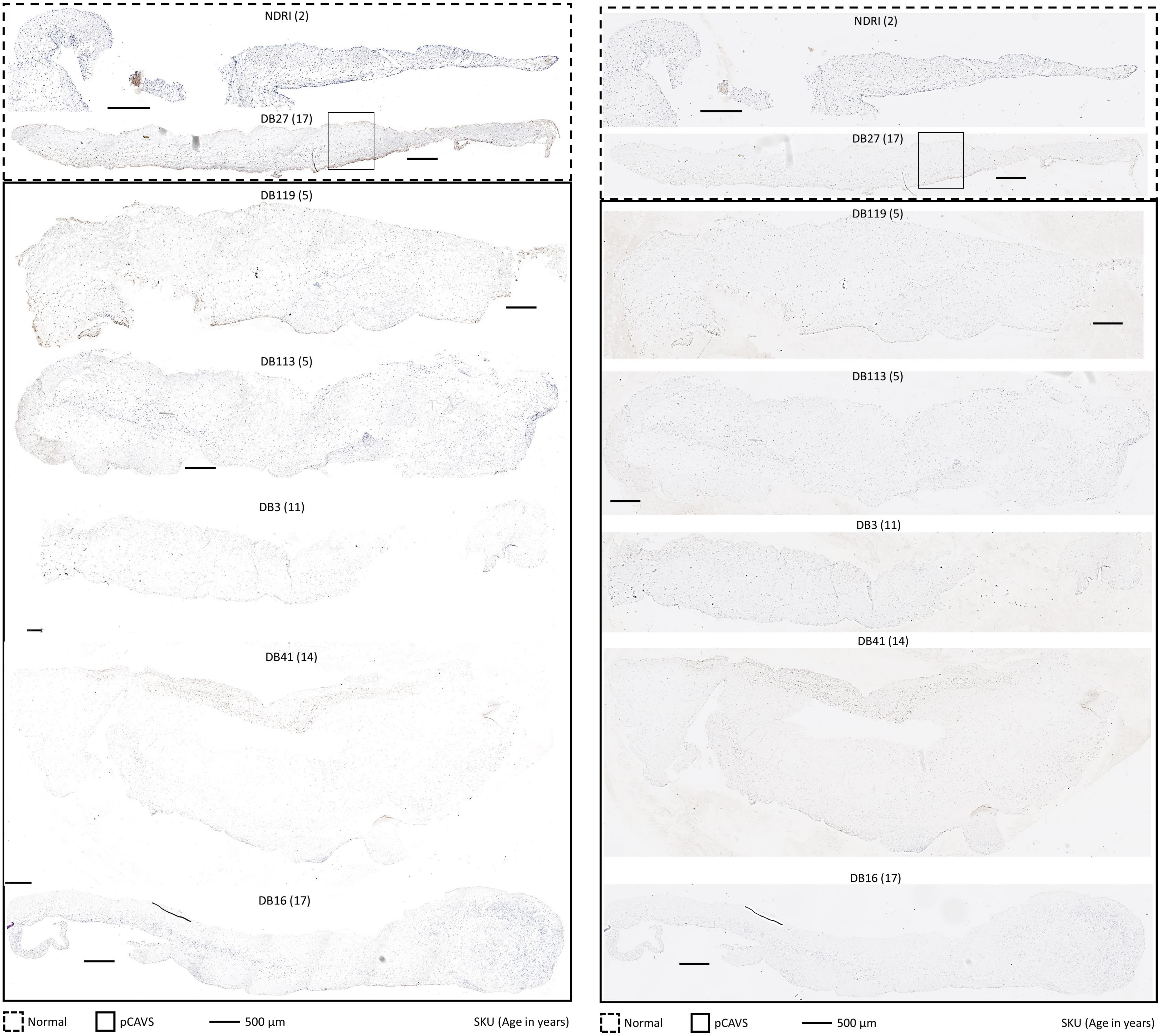

Supplemental Figure S6: Immunohistochemistry images with primary antibody BAMBI (chromogen, brown). Mayer's hematoxylin (blue) was used as a counterstain. Background subtracted samples are shown on the left, with raw images shown on the right.

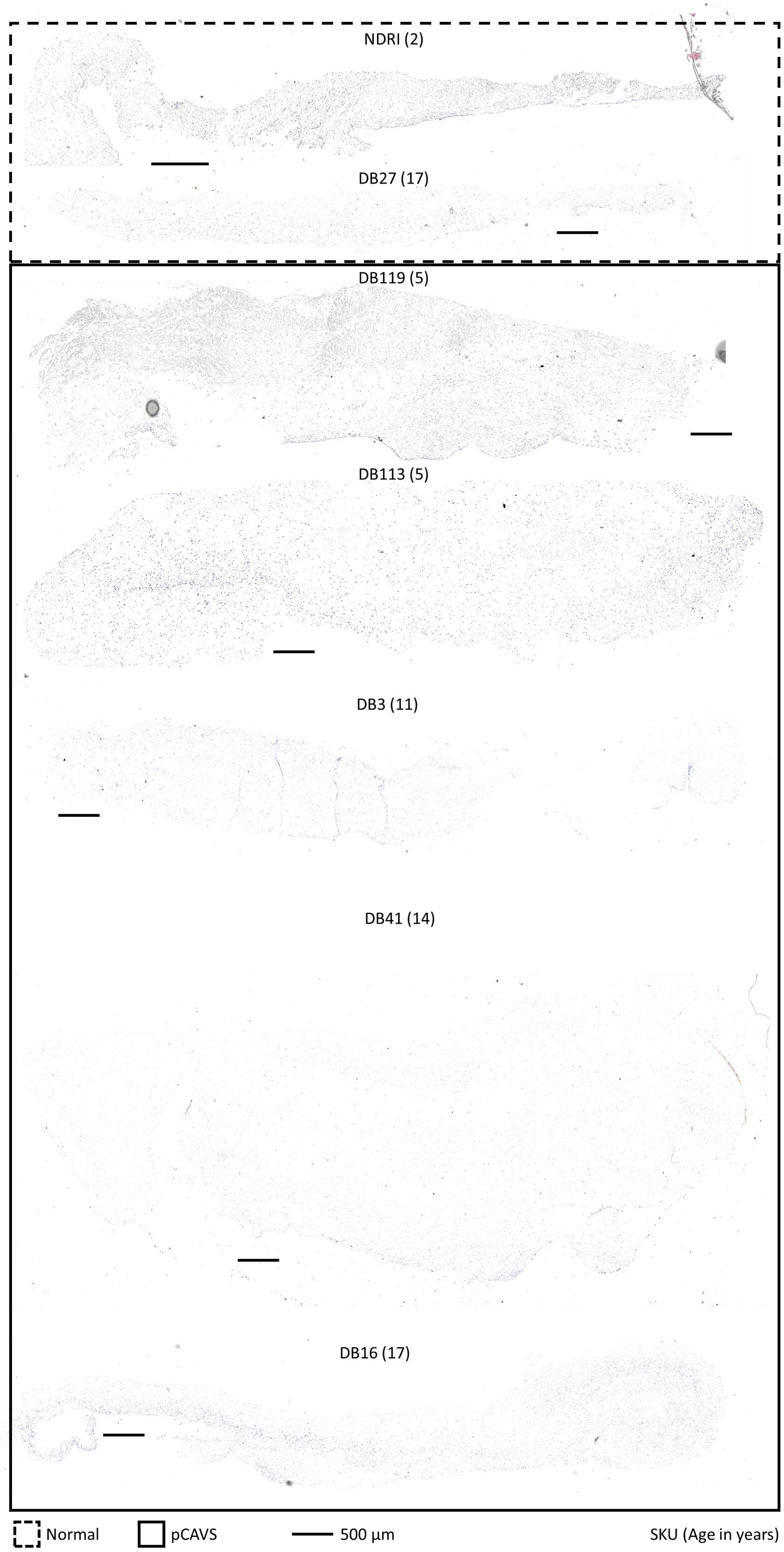

Supplemental Figure S7: Immunohistochemistry images of negative controls corresponding to images in Supplementary Fig. S6. No primary antibody was used. Mayer's hematoxylin (blue) was used as a counterstain.

Supplementary Table S1: Normal peptides identified with a LOG2(Fold Change) intensity value greater than 2 compared to pCAVS. PEP: Posterior error probability.

Protein Name	Uniprot Accession Number	Uniprot Name	Gene Name	Sequence	Oxidation (P)	Oxidation (P) Probabilities	Charges	Mass	PEP	MQ Peptide Score	Intensity	MS/MS Count	Normal Log Transform Mean	pCAVS Log Transform Mean	AVI Log Transform Mean
Basement membrane-specific heparan sulfate proteo	P98160	PGBM_HUMAN	HSPG2	LVNFTRSIEYSPQLEDAGSRE	0		3	2411.1605	0.000	122.8	2.E+07	4	2.325	-0.505	0.617
Basement membrane-specific heparan sulfate proteo	P98160	PGBM_HUMAN	HSPG2	NPSVQGGQCLPETNQAPL	1	NP(0.5)SVQGGQCLP(0.014)ETNQAP(0.486)L	2	1885.8364	0.034	81.7	3.E+06	1	1.347	-1.029	NaN
Biglycan	P21810	PGS1_HUMAN	BGN	LHSNNITKVG	0		2	1082.5720	0.088	88.8	4.E+05	1	-1.762	-4.009	-2.481
Collagen alpha-1(I) chain	P02452	CO1A1_HUMAN	COL1A1	FLPQPPQEKAHDGGR	1	FLP(0.004)QP(0.195)P(0.801)QEKAHDGGR	2;3	1691.8380	0.025	87.7	2.E+07	4	1.623	-0.460	-0.201
Collagen alpha-1(I) chain	P02452	CO1A1_HUMAN	COL1A1	GKDGLNGLP	0		2	870.4447	0.027	108.5	4.E+06	5	1.671	-0.993	-2.153
Collagen alpha-1(I) chain	P02452	CO1A1_HUMAN	COL1A1	GSPGENGAPGQMGPR	1	GSP(0.5)GENGAP(0.5)GQMGPR	2	1444.5889	0.014	97.5	3.E+05	4	-1.081	-3.205	NaN
Collagen alpha-1(I) chain	P02452	CO1A1_HUMAN	COL1A1	IAGAPGFPGAR	1	IAGAP(0.5)GFP(0.5)GAR	2	1044.5352	0.007	111.8	6.E+06	10	1.257	-0.737	-1.330
Collagen alpha-1(I) chain	P02452	CO1A1_HUMAN	COL1A1	PQPPQEKAHDGGR	0		3	1415.6906	0.162	83.1	8.E+05	1	1.076	-5.239	NaN
Collagen alpha-1(III) chain	P02461	CO3A1_HUMAN	COL3A1	ADGVPGKDGPR	0		2	1067.5360	0.078	85.3	1.E+05	1	-1.483	-4.338	NaN
Collagen alpha-1(III) chain	P02461	CO3A1_HUMAN	COL3A1	GEPGRDGNPGSDGLPGRDGSP	4	GEP(1)GRDGNP(1)GSDGLP(1)GRDGSP(1)	3	2056.8682	0.000	118.7	2.E+06	7	0.687	-2.264	-4.536
Collagen alpha-1(III) chain	P02461	CO3A1_HUMAN	COL3A1	GEPGRDGNPGSDGLPGRDGSP	1	GEP(0.25)GRDGNP(0.25)GSDGLP(0.25)GRDGSP(0.25)	3	2056.8682	0.000	133.9	1.E+06	3	1.250	-1.104	NaN
Collagen alpha-1(III) chain	P02461	CO3A1_HUMAN	COL3A1	GGPGADGVPGKDGPR	1	GGP(1)GADGVPGKDGPR	3	1351.6480	0.017	88.0	2.E+06	4	0.231	-3.189	-4.848
Collagen alpha-1(III) chain	P02461	CO3A1_HUMAN	COL3A1	GLKGENGLPGENGAPGPM	1	GLKGENGLP(0.5)GENGAP(0.25)GP(0.25)M	2	1743.7621	0.000	234.8	6.E+06	18	2.095	-0.397	NaN
Collagen alpha-1(IV) chain	P02462	CO4A1_HUMAN	COL4A1	QPGPKGDPGI	2	QP(1)GP(0.999)KGDP(0.001)GI	1	996.4876	0.091	88.4	1.E+06	4	1.678	-1.958	-0.703
Collagen alpha-1(V) chain	P20908	CO5A1_HUMAN	COL5A1	GETGDVGQMGPP	1	GETGDVGQMGP(0.224)P(0.776)	2	1175.4765	0.048	91.3	2.E+05	1	-1.680	-4.722	NaN
Collagen alpha-1(V) chain	P20908	CO5A1_HUMAN	COL5A1	GLQGPVGLP	1	GLQGPVGLP(1)	1	852.4705	0.118	83.6	1.E+07	3	2.498	0.273	0.355
Collagen alpha-1(VI) chain	P12109	CO6A1_HUMAN	COL6A1	FLTAGRTDPAHDVR	0		3	1554.7903	0.122	100.2	4.E+06	2	0.523	-1.661	-1.499
Collagen alpha-1(VI) chain	P12109	CO6A1_HUMAN	COL6A1	GEAGDEGNPGPDGAPGERGGPG	1	GEAGDEGNP(0.001)GP(0.005)DGAP(0.496)GERGGP(0.498)G	3	2265.9483	0.044	89.7	1.E+05	2	-1.934	-4.896	NaN
Collagen alpha-1(VI) chain	P12109	CO6A1_HUMAN	COL6A1	LTAGRTDPAHDVR	0		3	1407.7219	0.033	100.4	6.E+06	6	0.524	-1.646	-0.103
Collagen alpha-1(VI) chain	P12109	CO6A1_HUMAN	COL6A1	VAVVQYSGTGQQRPERA	0		2;3	1844.9493	0.033	80.9	1.E+07	7	2.550	-0.394	1.859
Collagen alpha-1(XI) chain	P12107	COBA1_HUMAN	COL11A1	MGPQGEPGPPGQQGN	1	MGP(0.004)QGEP(0.831)GP(0.134)P(0.032)GQQGN	2	1466.6096	0.060	76.3	6.E+06	3	1.044	-1.134	NaN
Collagen alpha-1(XVI) chain	Q07092	COGA1_HUMAN	COL16A1	GLAGEPGP	0		1	696.3443	0.143	77.7	4.E+05	1	-0.018	-3.272	NaN
Collagen alpha-1(XVIII) chain	P39060	COIA1_HUMAN	COL18A1	GPPGQFPFDFLQ	1	GP(0.5)P(0.5)GQFPFDFLQ	2	1364.6401	0.011	119.7	1.E+06	1	0.563	-4.107	-1.940
Collagen alpha-1(XVIII) chain	P39060	COIA1_HUMAN	COL18A1	GRFGVNSSDVPGPA	0		2	1359.6419	0.027	103.2	8.E+05	1	1.275	-2.657	NaN
Collagen alpha-2(I) chain	P08123	CO1A2_HUMAN	COL1A2	GARGSDGSVGPV	0		2	1057.5152	0.002	134.2	6.E+06	18	1.669	-1.130	-2.907
Collagen alpha-2(I) chain	P08123	CO1A2_HUMAN	COL1A2	GAVGSPGVNGAPGEA	2	GAVGSP(1)GVNGAP(1)GEA	1;2	1271.5630	0.000	166.1	1.E+07	34	2.111	-0.500	-2.597
Collagen alpha-2(I) chain	P08123	CO1A2_HUMAN	COL1A2	GAVGSPGVNGAPGEAGRDGNP	3	GAVGSP(1)GVNGAP(1)GEAGRDGNP(1)	2	1883.8246	0.000	126.1	8.E+05	11	-0.781	-3.090	-4.866
Collagen alpha-2(I) chain	P08123	CO1A2_HUMAN	COL1A2	GSPGVNGAPGEAGRDGNPGNDG	3	GSP(1)GVNGAP(1)GEAGRDGNP(0.012)GNDGP(0.494)P(0.494	2	2193.9159	0.000	113.3	1.E+05	2	-2.024	-4.749	NaN
Collagen alpha-2(I) chain	P08123	CO1A2_HUMAN	COL1A2	GSPGVNGAPGEAGRDGNPGNDG	2	GSP(0.5)GVNGAP(0.5)GEAGRDGNP(0.492)GNDGP(0.254)P(0.	2	2209.9108	0.000	145.7	9.E+05	13	-1.047	-2.995	NaN
Collagen alpha-2(I) chain	P08123	CO1A2_HUMAN	COL1A2	LVGEPGPA	1	LVGEP(1)GPA	1	754.3861	0.048	106.8	9.E+06	21	1.705	-0.824	-0.352
Collagen alpha-2(I) chain	P08123	CO1A2_HUMAN	COL1A2	PGVNGAPGEA	2	GSP(0.5)GVNGAP(0.5)GEAGRDGNP(0.492)GNDGP(0.254)P(0.	1	900.3825	0.036	99.1	5.E+05	4	-0.046	-2.481	NaN
Collagen alpha-2(I) chain	P08123	CO1A2_HUMAN	COL1A2	SPGVNGAPGEA	2	SP(1)GVNGAP(1)GEA	1	987.4145	0.095	82.7	1.E+05	1	-2.541	-4.824	NaN
Collagen alpha-2(VI) chain	P12110	CO6A2_HUMAN	COL6A2	LANMTEQIR	0		2	1075.5332	0.016	88.2	3.E+06	2	-0.565	-2.723	-1.652
Collagen alpha-2(VI) chain	P12110	CO6A2_HUMAN	COL6A2	LDGSERLGEQN	0		2	1216.5684	0.021	99.8	2.E+05	3	-1.145	-4.243	-5.432
Collagen alpha-2(VI) chain	P12110	CO6A2_HUMAN	COL6A2	LYRNDYAT	0		2	1015.4611	0.134	79.7	2.E+06	1	1.298	-2.462	-3.148
Collagen alpha-3(VI) chain	P12111	CO6A3_HUMAN	COL6A3	FDFDEYQPEMLEKFR	0		2;3	2008.8877	0.005	112.4	6.E+07	29	3.658	1.615	1.560
Collagen alpha-3(VI) chain	P12111	CO6A3_HUMAN	COL6A3	HANTKVGLEHLR	0		3	1373.7528	0.052	89.4	4.E+05	2	-2.426	-5.142	-5.191
Collagen alpha-3(VI) chain	P12111	CO6A3_HUMAN	COL6A3	PEVPRPQAAKPA	3	P(1)EVP(1)RP(1)QAAKPA	2	1307.6834	0.065	84.2	3.E+06	2	2.204	-1.657	NaN
Collagen alpha-3(VI) chain	P12111	CO6A3_HUMAN	COL6A3	PLRLNLLDLDYE	0		2	1472.7875	0.002	120.3	7.E+05	1	0.886	-1.794	-2.687
Dentin sialophosphoprotein	Q9NZW4	DSPP_HUMAN	DSPP	RSNTNGNTDKNTQNGDV	0		2	1838.7402	0.022	88.3	2.E+06	2	0.426	-1.956	NaN
Fibromodulin	Q06828	FMOD_HUMAN	FMOD	LHLDHNQISR	0		3	1231.6422	0.074	106.4	3.E+06	4	0.752	-1.649	-1.853
Fibromodulin	Q06828	FMOD_HUMAN	FMOD	LRELHLDHNQ	0		3	1273.6527	0.138	90.1	2.E+06	2	-0.259	-2.861	-4.221
Fibromodulin	Q06828	FMOD_HUMAN	FMOD	LYLDHNNLTR	0		2;3	1258.6306	0.011	111.1	1.E+07	6	2.403	0.070	-0.457
Fibronectin	P02751	FINC_HUMAN	FN1	NLAPDSSSVVVSG	1	NLAP(1)DSSSVVVSG	2	1247.5881	0.062	81.3	4.E+06	4	1.811	-1.432	-2.549
Tenascin-X	P22105	TENX_HUMAN	TNXB	LLEETTVRTE	0		2	1189.6190	0.016	105.5	2.E+06	2	-0.110	-2.022	-1.880
Vimentin	P08670	VIME_HUMAN	VIM	KNLQEAEEWYKSK	0		3	1651.8206	0.000	161.2	8.E+06	12	1.368	-0.746	-0.918

Supplemental Table S2: pCAVS peptides identified with a LOG2(Fold Change) intensity value greater than 2 compared to Normal. PEP: Posterior error probability.

Protein Name	Uniprot Accession Number	Uniprot Name	Gene Name	Sequence	Oxidation (P)	Oxidation (P) Probabilities	Charges	Mass	PEP	MQ Peptide Score	Intensity	MS/MS Count	Normal Log Transform Mean	pCAVS Log Transform Mean	AVI Log Transform Mean
Asporin	Q9BXN1	ASPN_HUMAN	ASPN	SEIPLNLPKS	0		1	1096.6128	0.007	120.3	4.E+06	7	-2.1048	0.1160	NaN
Basement membrane-specific heparan sulfate proteoglycan core protein	Q15582	BGH3_HUMAN	BGH3	LAPTNEAFEKIPSETLNR	0		2;3	2029.0480	0.040	79.8	7.E+07	6	0.1578	2.4777	1.1328
Biglycan	P21810	PGS1_HUMAN	BCN	IELEDLLRYSKLYR	0		3	1809.9989	0.092	73.8	1.E+07	2	-0.9563	1.4436	-0.9051
Biglycan	P21810	PGS1_HUMAN	BCN	LARVPSGLPDLK	0		2;3	1264.7503	0.003	136.3	7.E+07	38	-0.5734	1.6439	1.6777
Collagen alpha-1(I) chain	P02452	CO1A1_HUMAN	COL1A1	GLPGTAGLP	2	GLP(1)GTAGLP(1)	1;2	813.4232	0.009	126.2	2.E+08	21	0.8426	4.3823	-0.9657
Collagen alpha-1(I) chain	P02452	CO1A1_HUMAN	COL1A1	PPQEKAHDGGRYY	0		2	1516.7059	0.049	85.8	8.E+07	5	-0.2527	2.1825	1.0390
Collagen alpha-1(III) chain	P02461	CO3A1_HUMAN	COL3A1	FAPYYG	0		1	716.3170	0.158	77.5	6.E+07	2	-1.6375	2.8232	1.1229
Collagen alpha-1(III) chain	P02461	CO3A1_HUMAN	COL3A1	GAPGLKGENGLP	1	GAP(0.5)GLKGENGLP(0.5)	2	1125.5666	0.029	96.1	8.E+07	11	-3.4981	3.7819	2.7979
Collagen alpha-1(III) chain	P02461	CO3A1_HUMAN	COL3A1	GLAGTAGEPGRDGNPGSDGLP	3	GLAGTAGEP(1)GRDGNP(1)GSDGLP(1)	2	1942.8504	0.000	123.1	2.E+06	1	-2.2755	-0.1059	NaN
Collagen alpha-1(III) chain	P02461	CO3A1_HUMAN	COL3A1	GLKGENGLP	1	GLKGENGLP(1)	2	900.4553	0.029	107.3	1.E+07	9	-3.1905	1.1839	0.2164
Collagen alpha-1(III) chain	P02461	CO3A1_HUMAN	COL3A1	GQPGVMGFP	2	GQP(1)GVMGFP(1)	1;2	936.4011	0.017	118.0	8.E+07	19	1.0131	3.3746	-0.4589
Collagen alpha-1(III) chain	P02461	CO3A1_HUMAN	COL3A1	GQPGVMGFP	1	GQPGVMGFP(1)	1;2	936.4011	0.020	113.1	1.E+07	10	-1.1158	2.2727	-0.0280
Collagen alpha-1(III) chain	P02461	CO3A1_HUMAN	COL3A1	GRPGRPGERGLPGPPGIKGPA	1	GRP(0.061)GRP(0.935)GERGLP(0.003)GP(0.001)PGIKGPA	2	2038.1184	0.056	78.5	9.E+06	1	-1.7151	0.3776	0.0850
Collagen alpha-1(III) chain	P02461	CO3A1_HUMAN	COL3A1	LAGTAGEPGRDGNPGSDGLP	1	LAGTAGEP(0.5)GRDGNPGSDGLP(0.5)	2	1868.8500	0.000	132.2	2.E+07	32	-1.5434	2.5790	0.1992
Collagen alpha-1(III) chain	P02461	CO3A1_HUMAN	COL3A1	VAVGGLAGYPGP	1	VAVGGLAGYP(0.5)GP(0.5)	1	1088.5502	0.148	71.7	2.E+07	1	-2.1409	-0.0839	2.2532
Collagen alpha-1(IV) chain	P02462	CO4A1_HUMAN	CO4A1	DGLPGDMGPPG	0		1	1011.4331	0.168	73.7	2.E+07	1	-0.8143	1.6616	-1.7123
Collagen alpha-1(V) chain	P20908	CO5A1_HUMAN	COL5A1	GAPGKPGPDGL	1	GAP(0.5)GKP(0.452)GP(0.048)DGL	1	980.4927	0.058	90.7	1.E+07	12	-1.3273	1.2474	0.0534
Collagen alpha-1(V) chain	P20908; P12107	CO5A1_HUMAN; COBA1_HUMAN	COL5A1; COL11A1	GPPGTMLMLP	1	GP(0.5)P(0.5)GTMLMLP	1;2	1044.4984	0.000	138.4	5.E+07	40	-1.5024	1.2157	0.5055
Collagen alpha-1(V) chain	P20908	CO5A1_HUMAN	COL5A1	GSIGFPGFP	2	GSIGFP(1)GFP(1)	1	909.4232	0.030	106.8	6.E+06	7	0.5141	2.8417	-0.7466
Collagen alpha-1(VI) chain	P12109	CO6A1_HUMAN	COL6A1	IDTIVDMIKNNVEQV	0		2	1729.8920	0.000	178.7	3.E+06	6	-2.8423	-0.5475	-2.4121
Collagen alpha-1(VI) chain	P12109	CO6A1_HUMAN	COL6A1	LVITDGRSDTQRDTTPLNV	0		2;3	2100.0811	0.037	59.2	4.E+07	2	-1.0554	1.2432	1.4888
Collagen alpha-1(VI) chain	P12109	CO6A1_HUMAN	COL6A1	QYSHSQMQEHVS	0		2	1459.6150	0.048	52.7	1.E+07	8	-2.6176	-0.0316	-0.5003
Collagen alpha-1(XIV) chain	Q05707	COEA1_HUMAN	COL14A1	VFTTEEVPAQQYLEIDEVTTDSFR	0		2;3	2816.3392	0.000	166.7	6.E+06	11	-2.4274	-0.3436	-0.8350
Collagen alpha-2(I) chain	P08123	CO1A2_HUMAN	COL1A2	GARGLPGERGRVGAPGP	1	GARGLP(1)GERGRVGAPGP	2	1618.8652	0.113	69.3	1.E+08	1	0.7930	2.8586	0.4042
Collagen alpha-2(I) chain	P08123	CO1A2_HUMAN	COL1A2	GPSGPSGLPGER	1	GPSGPSGLP(1)GER	2	1125.5415	0.028	97.8	4.E+05	1	-2.7069	-0.1752	-4.5097
Collagen alpha-2(I) chain	P08123	CO1A2_HUMAN	COL1A2	GSPGNIGPAGKEGPVGLPGIDGRP	3	GSP(1)GNIGPAGKEGP(0.002)VGLP(0.998)GIDGRP(1)	3	2246.1291	0.064	86.0	5.E+06	1	-3.0070	-0.1115	NaN
Collagen alpha-2(I) chain	P08123	CO1A2_HUMAN	COL1A2	GVRGPNGDAGRPGEPGLM	2	GPAGVRGPNGDAGRP(1)GEP(1)GLM	2;3	1784.8112	0.019	91.1	5.E+06	3	-2.9732	-0.3157	-1.1215
Collagen alpha-2(I) chain	P08123	CO1A2_HUMAN	COL1A2	SGGGYDFGYDGDFYRA	0		2	1745.6958	0.000	145.2	1.E+07	14	-1.6850	0.7243	NaN
Collagen alpha-2(I) chain	P08123	CO1A2_HUMAN	COL1A2	VGLGPGPMG	0		1	799.3898	0.152	77.9	2.E+06	1	-2.5026	-0.2940	NaN
Collagen alpha-2(V) chain	P05997	CO5A2_HUMAN	COL5A2	GPVGSPGLP	3	GP(1)VGSP(1)GLP(1)	1	827.4025	0.091	89.7	6.E+07	6	3.2119	5.2425	NaN
Collagen alpha-2(VI) chain	P12110	CO6A2_HUMAN	CO6A2	FSHVGAG	0		1	673.3184	0.036	104.9	1.E+06	3	-3.8602	-1.1458	NaN
Collagen alpha-2(VI) chain	P12110	CO6A2_HUMAN	CO6A2	LGSDVDMDVLTT	0		1;2	1280.5806	0.013	111.0	8.E+07	40	0.2976	2.6329	1.2369
Collagen alpha-2(VI) chain	A6NMZ7	CO6A6_HUMAN	CO6A6	LLNANLLDS	0		1	971.5288	0.031	106.4	1.E+05	2	-3.1398	-0.8015	NaN
Collagen alpha-2(VI) chain	P12110	CO6A2_HUMAN	CO6A2	VTMQSPTDILL	0		1;2	1216.6373	0.013	104.0	3.E+07	10	-2.1749	0.9699	-0.0771
Collagen alpha-3(VI) chain	P12111	CO6A3_HUMAN	COL6A3	AAEGIPKLLVL	0		2	1122.7012	0.033	95.5	9.E+06	5	-2.9678	0.2197	0.8449
Collagen alpha-3(VI) chain	P12111	CO6A3_HUMAN	COL6A3	FMYDRPLRLNLLDLDYE	1	FMYDRP(1)LRLNLLDLDYE	3	2201.0827	0.023	87.5	3.E+06	1	-2.1313	0.3091	NaN
Collagen alpha-3(VI) chain	P12111	CO6A3_HUMAN	COL6A3	FRELPSLEQKL	0		2;3	1358.7558	0.000	147.7	4.E+08	61	1.1809	3.2767	4.2496
Collagen alpha-3(VI) chain	P12111	CO6A3_HUMAN	COL6A3	KSVLLDKIKNLQVA	0		2;3	1567.9661	0.100	73.1	4.E+07	2	-0.6385	2.7378	1.6081
Collagen alpha-3(VI) chain	P12111	CO6A3_HUMAN	COL6A3	LGGPTPNTGAALEFVLRN	0		2	1825.9686	0.000	193.5	1.E+07	10	-1.6325	0.5093	0.4463
Collagen alpha-3(VI) chain	P12111	CO6A3_HUMAN	COL6A3	LQPVLQPLPSPGVGGKRDVVF	0		2	2202.2525	0.000	117.1	6.E+06	7	-3.0584	-0.7657	-0.2520
Collagen alpha-3(VI) chain	P12111	CO6A3_HUMAN	COL6A3	LRLNLLDLDYE	0		2	1376.7187	0.000	138.0	3.E+05	2	-4.3541	0.9138	-3.1668
Collagen alpha-3(VI) chain	P12111	CO6A3_HUMAN	COL6A3	LTEDTLKVYLNK	0		2;3	1435.7922	0.026	102.5	2.E+07	3	-2.0618	0.1143	-0.3290
Collagen alpha-3(VI) chain	P12111	CO6A3_HUMAN	COL6A3	LTLLGGPTPNTGAALEFVLRN	0		2	2153.1845	0.000	191.6	4.E+06	7	-2.9610	-0.6990	-0.4187
Collagen alpha-3(VI) chain	P12111	CO6A3_HUMAN	COL6A3	LVLPAGRDLTDFLENV	0		2	1770.9516	0.001	122.3	2.E+07	30	-1.8448	0.5836	-1.6825
Collagen alpha-3(VI) chain	P12111	CO6A3_HUMAN	COL6A3	PGEDGYRGYPGDEGGPGER	1	GIPGEDGYRGYP(0.967)GDEGGP(0.033)GER	2;3	1979.8246	0.046	79.3	6.E+06	5	-1.7773	0.2732	NaN
Collagen alpha-3(VI) chain	P12111	CO6A3_HUMAN	COL6A3	PGVGGKRDVVF	0		1	1129.6244	0.131	78.1	4.E+06	1	-3.7122	-0.5140	-1.4834
Collagen alpha-3(VI) chain	P12111	CO6A3_HUMAN	COL6A3	VAQKGFESKVDAILNR	0		2;3	1773.9737	0.000	139.5	2.E+08	15	-1.8115	2.7017	3.0137
Collagen alpha-3(VI) chain	P12111	CO6A3_HUMAN	COL6A3	VLQPLPSPGVGG	0		1;2	1119.6288	0.000	157.3	4.E+07	32	-0.8885	1.4184	-0.2886
Collagen alpha-3(VI) chain	P12111	CO6A3_HUMAN	COL6A3	VREFRELPNIEERIMNS	0		2;3	2131.0844	0.000	113.1	1.E+08	10	1.6446	3.9760	2.2189
Decorin	P07585	PGS2_HUMAN	DCN	LHLDNNKLTRVPGG	0		3	1532.8423	0.007	125.5	2.E+07	5	-1.1238	1.1035	-0.3927
Decorin	P07585	PGS2_HUMAN	DCN	LILVNNKISKVSPGA	0		2;3	1551.9348	0.036	82.7	3.E+07	5	-1.3148	1.1352	-0.3433
Fibromodulin	P35555	FBN1_HUMAN	FBN1	LGPIPPVLPVPPGFPP	0		2	1592.9330	0.002	109.3	2.E+07	17	-2.7646	-0.6033	-1.0830
Fibromodulin	P35555	FBN1_HUMAN	FBN1	SPNISACIDINECEL	0		2	1620.7011	0.090	72.3	6.E+06	2	-2.0677	0.5099	NaN
Lumican	P51884	LUM_HUMAN	LUM	IARLPSGLP	0		2	922.5600	0.114	84.6	2.E+07	1	-1.9802	0.7323	0.3523
Lumican	P51884	LUM_HUMAN	LUM	IARLPSGLPVSL	0		2	1221.7445	0.012	119.5	8.E+07	20	-0.8069	1.2787	1.5403
Lumican	P51884	LUM_HUMAN	LUM	KNIPTVNENLENYY	0		2	1710.8101	0.022	110.1	2.E+07	5	-2.1286	0.1970	-1.0631
Lumican	P51884	LUM_HUMAN	LUM	LSFNQIARLPSGLPVSL	0		2	1811.0305	0.036	79.9	2.E+06	1	-4.5068	-1.2079	-3.8748
Microfibril-associated glycoprotein 4	P55083	MFAP4_HUMAN	MFAP4	LIYPSGPSVPVP	0		1;2	1224.6754	0.002	145.6	9.E+07	29	0.0929	2.3167	1.2361
Serpin H1	P50454	SERPH_HUMAN	SERPHIN	RSALQSINEWA	0		2	1275.6095	0.030	96.0	6.E+07	4	-4.1829	5.4669	4.4257
Tenascin	P24821	TENA_HUMAN	TNC	IATVDSYVISYTGEKVPEITR	0		3	2340.2213	0.173	69.1	9.E+06	1	-2.4106	1.4159	-0.9055
Tenascin	P24821	TENA_HUMAN	TNC	IKDVPGDRTTIDLTEDENQYS	0		2;3	2408.1343	0.000	90.9	2.E+07	5	-1.4715	0.6487	0.7481
Tenascin	P24821	TENA_HUMAN	TNC	NKKSIPVSARVATY	0		2	1533.8515	0.121	71.0	9.E+07	1	-0.2405	1.9431	1.6421
Tenascin-X	P22105	TENX_HUMAN	TNXB	IQFIPTTEGASPPFTARVPSS	0		2	2202.1321	0.000	163.9	1.E+07	11	-1.5615	0.6085	-0.7754
Tenascin-X	P22105	TENX_HUMAN	TNXB	LLVDGDQSKILISGLEPSTPYRF	0		3	2547.3585	0.002	101.6	3.E+06	2	-1.7289	0.7801	NaN
Tenascin-X	P22105	TENX_HUMAN	TNXB	MRVPEGPGAHEEVLPGDVRQA	0		3	2243.1117	0.004	108.1	1.E+07	4	-1.8627	0.7799	0.7046
Tenascin-X	P22105	TENX_HUMAN	TNXB	WTVPEGQFDHF	0		1;2	1361.6041	0.000	160.8	2.E+08	43	0.4038	2.9801	2.2208
Vimentin	P08670	VIME_HUMAN	VIM	AKNLQEAEEWYKSK	0		2;3	1722.8577	0.000	156.0	2.E+07	11	-2.1688	0.7391	-0.6449
Vimentin	P08670	VIME_HUMAN	VIM	FANYIDKVRFLEQQNK	0		3	2012.0480	0.003	104.9	5.E+07	6	-0.1309	2.1718	2.8007
Vimentin	P08670	VIME_HUMAN	VIM	FANYIDKVRFLEQQNKIL	0		2;3	2238.2161	0.002	68.0	7.E+07	4	0.5245	3.0872	2.5203
Vimentin	P08670	VIME_HUMAN	VIM	LKGQGKSRLGDLYEEEMRE	0		2;3;4	2237.1110	0.000	114.2	1.E+08	13	-0.1345	2.6271	2.3966
Vimentin	P08670	VIME_HUMAN	VIM	LLEGEESRISLPLPNF	0		2	1812.9622	0.024	83.6	1.E+06	3	-3.7843	-0.5447	NaN
Vimentin	P08670	VIME_HUMAN	VIM	LLEGEESRISLPLPNFSS	0		2	1988.0102	0.026	86.3	1.E+07	9	-2.0795	0.4440	1.1336
Vimentin	P08670	VIME_HUMAN	VIM	LREKLQEEMLQREEAENT	0		3	2261.0958	0.021	89.5	2.E+08	5	1.5791	3.9456	4.2966
Vimentin	P08670	VIME_HUMAN	VIM	QEEIAFL	0		1	848.4280	0.045	88.6	3.E+07	6	-2.0461	2.5316	0.9145

Supplementary Table S3: Proteins identified via Collagenase Type III LC-MSMS Proteomics. MWU: Mann Whitney U-Test

Protein Name	Protein Accession Numbers (Uniprot)	Gene Symbol	Normal Peptide counts (all)	pCAVS Peptide counts (all)	AVI Peptide counts (all)	Normal Peptide Counts (unique)	pCAVS Peptide Counts (unique)	AVI Peptide Counts (unique)	Normal Sequence Coverage [%]	pCAVS Sequence Coverage [%]	AVI Sequence Coverage [%]	Normal LOG2 Transform Intensity	pCAVS LOG2 Transform Intensity	AVI LOG2 Transform Intensity	Log2(FC) (pCAVS/N)	Log2(FC) (AVI/N)	Log2(FC) (CAVS/AVI)	Norm-pCAVS MWU p value	Normal-AVI MWU p value	pCAVS-AVI MWU p value
Collagen alpha-6(VI) chain	sp|A6NMZ7|CO6A6	COL6A6	57	170	56	57	170	56	7.0	7.9	5.5	19.87	19.06	19.51	-0.81	-0.36	-0.45	0.226	0.730	0.510
Collagen alpha-1(I) chain	sp|P02452|CO1A1	COL1A1	307	1049	266	303	1035	264	23.0	29.6	19.4	26.36	26.34	25.11	-0.02	-1.25	1.23	0.949	0.016	0.001
Collagen alpha-1(III) chain	sp|P02461|CO3A1	COL3A1	196	687	167	130	437	99	21.5	26.8	18.3	23.74	23.37	22.58	-0.37	-1.16	0.79	0.661	0.063	0.180
Collagen alpha-1(IV) chain	sp|P02462|CO4A1	COL4A1	90	229	73	90	227	71	13.4	14.8	9.5	21.80	21.00	21.16	-0.80	-0.64	-0.16	0.343	0.413	1.000
Prelamin-A/C	sp|P02545|LMNA	LMNA	11	48	20	11	48	20	6.6	9.7	8.7	17.01	17.10	17.59	0.09	0.58	-0.49	1.000	1.000	0.165
Complement C1q subcomponent subunit A	sp|P02745|C1QA	C1QA	16	51	8	16	51	8	12.3	13.1	5.1	17.17	17.37	18.03	0.19	0.86	-0.66	0.851	1.000	0.513
Fibronectin	sp|P02751|FINC	FN1	50	334	95	50	334	95	6.4	14.5	9.7	19.77	21.35	21.92	1.59	2.15	-0.57	0.040	0.032	0.320
Vitronectin	sp|P04004|VTNC	VTN	9	45	5	2	17	1	4.4	8.3	1.9	0.00	17.25	0.00	17.25	0.00	17.25	UTC	UTC	UTC
Collagen alpha-2(V) chain	sp|P05997|CO5A2	COL5A2	81	323	61	81	322	61	11.9	15.9	7.7	22.56	22.51	21.59	-0.05	-0.97	0.92	0.949	0.032	0.005
Decorin	sp|P07585|PGS2	DCN	112	392	89	112	390	89	43.0	52.4	41.4	21.72	22.17	21.53	0.45	-0.19	0.64	0.226	0.413	0.180
Collagen alpha-2(I) chain	sp|P08123|CO1A2	COL1A2	487	1538	380	485	1533	379	33.9	37.7	28.2	26.23	26.09	25.28	-0.14	-0.95	0.81	1.000	0.016	0.001
Collagen alpha-2(IV) chain	sp|P08572|CO4A2	COL4A2	68	136	55	68	134	54	6.9	6.1	5.4	20.66	19.91	19.96	-0.75	-0.70	-0.05	0.489	0.556	0.827
Vimentin	sp|P08670|VIME	VIM	300	1224	456	294	1196	446	72.6	83.2	83.9	23.95	24.37	24.68	0.42	0.73	-0.31	0.489	0.190	0.827
SPARC	sp|P09486|SPRC	SPARC	4	18	2	4	18	2	5.8	9.2	1.7	0.00	16.67	0.00	16.67	0.00	16.67	UTC	UTC	UTC
Collagen alpha-1(XI) chain	sp|P12107|COBA1	COL11A1	57	202	45	18	85	19	6.7	9.2	5.2	20.31	19.58	18.84	-0.72	-1.47	0.74	0.190	0.667	0.333
Collagen alpha-1(VI) chain	sp|P12109|CO6A1	COL6A1	256	944	262	254	936	261	37.5	45.1	31.2	24.37	24.20	24.20	-0.18	-0.17	0.00	0.226	0.286	0.913
Collagen alpha-2(VI) chain	sp|P12110|CO6A2	COL6A2	243	848	238	241	840	235	30.9	34.6	27.1	23.79	23.78	23.43	-0.02	-0.36	0.35	0.949	0.190	0.221
Collagen alpha-3(VI) chain	sp|P12111|CO6A3	COL6A3	724	2839	842	724	2834	842	32.0	38.9	31.8	25.23	25.62	25.86	0.39	0.63	-0.24	0.138	0.063	0.441
Collagen alpha-1(V) chain	sp|P20908|CO5A1	COL5A1	147	503	118	108	385	91	12.1	14.7	9.5	22.94	23.03	22.35	0.09	-0.59	0.68	0.753	0.063	0.027
Biglycan	sp|P21810|PGS1	BGN	207	722	235	207	720	235	44.0	50.9	43.5	23.78	23.88	24.29	0.10	0.50	-0.41	1.000	0.413	0.583
Tenascin-X	sp|P22105|TENX	TNXB	115	660	134	115	660	134	12.1	20.9	11.0	20.95	22.75	21.87	1.80	0.93	0.87	0.056	0.730	0.320
Fibulin-1	sp|P23142|FBLN1	FBLN1	5	20	9	5	20	6	3.0	4.2	2.9	16.80	17.88	0.00	1.08	-16.80	17.88	0.056	UTC	UTC
Tenascin	sp|P24821|TENA	TNC	18	163	43	18	163	43	2.7	8.9	5.0	18.95	19.77	19.65	0.81	0.69	0.12	0.225	0.143	0.827
Fibrillin-1	sp|P35555|FBN1	FBN1	25	113	32	25	113	32	2.1	2.5	1.5	19.85	20.89	21.03	1.03	1.18	-0.14	0.280	0.190	0.827
Myosin-10	sp|P35580|MYH10	MYH10	15	73	26	15	73	26	2.0	3.6	3.0	17.70	18.27	19.28	0.57	1.57	-1.01	1.000	0.533	0.412
Collagen alpha-1(XV) chain	sp|P39059|COFA1	COL15A1	46	139	41	46	139	41	8.0	8.4	5.1	20.28	19.85	19.79	-0.43	-0.49	0.06	0.753	0.413	0.827
Collagen alpha-1(XVIII) chain	sp|P39060|COIA1	COL18A1	35	39	15	35	39	15	6.9	2.9	2.4	19.97	18.37	18.96	-1.60	-1.01	-0.59	0.374	1.000	0.545
Cartilage oligomeric matrix protein	sp|P49747|COMP	COMP	16	69	15	12	56	14	5.5	9.0	5.3	18.48	18.85	18.82	0.37	0.34	0.03	0.769	1.000	0.949
Serpin H1	sp|P50454|SERPH	SERPINH	3	18	12	3	18	12	2.0	5.3	7.9	0.00	0.00	18.44	0.00	18.44	-18.44	UTC	UTC	UTC
Lumican	sp|P51884|LUM	LUM	103	418	130	60	271	86	38.0	42.0	35.8	22.24	22.40	22.40	0.17	0.17	0.00	0.489	0.556	0.827
Microfibril-associated glycoprotein 4	sp|P55083|MFAP4	MFAP4	62	171	43	62	171	43	31.0	34.3	21.3	20.59	20.93	20.05	0.34	-0.54	0.88	0.571	0.486	0.177
Basement membrane-specific heparan sulfa	sp|P98160|PGBM	HSPG2	159	422	161	159	422	161	8.7	8.7	7.3	21.68	20.90	21.80	-0.78	0.12	-0.90	0.280	0.905	0.377
Collagen alpha-1(XIV) chain	sp|Q05707|COEA1	COL14A1	52	159	90	52	159	90	8.7	9.1	11.2	19.82	19.09	21.39	-0.73	1.57	-2.30	0.280	0.286	0.090
Fibromodulin	sp|Q06828|FMOD	FMOD	121	443	104	117	430	103	44.3	50.7	34.3	21.64	21.65	21.11	0.00	-0.53	0.54	1.000	0.190	0.267
Collagen alpha-1(XVI) chain	sp|Q07092|COGA1	COL16A1	30	123	32	28	111	29	5.0	8.2	4.9	20.28	20.03	19.01	-0.25	-1.27	1.02	0.753	0.114	0.138
Dermatopontin	sp|Q07507|DERM	DPT	19	73	21	19	73	21	17.2	22.0	18.4	18.74	18.74	18.31	0.00	-0.43	0.42	0.641	0.133	0.280
Nidogen-2	sp|Q14112|NID2	NID2	11	50	17	11	50	17	2.1	3.5	2.4	17.35	17.87	18.03	0.52	0.67	-0.15	0.571	1.000	1.000
Transforming growth factor-beta-induced prc	sp|Q15582|BGH3	BGH3	47	277	70	47	277	70	15.7	29.9	19.2	19.44	21.28	21.08	1.84	1.64	0.21	0.018	0.190	0.441
Collagen alpha-1(XXI) chain	sp|Q96P44|COLA1	COL21A1	18	55	14	18	50	14	6.3	7.5	4.3	19.00	19.24	18.58	0.23	-0.42	0.65	0.489	0.057	0.126
Collagen alpha-1(XII) chain	sp|Q99715|COCA1	COL12A1	56	197	64	56	197	64	5.6	7.0	5.4	19.61	19.35	19.29	-0.25	-0.32	0.06	0.753	0.905	1.000
Asporin	sp|Q9BXN1|ASPN	ASPN	42	115	38	42	115	38	18.7	22.9	17.3	23.02	22.74	23.00	-0.28	-0.02	-0.26	0.571	1.000	0.743
Dentin sialophosphoprotein	sp|Q9NZW4|DSPP	DSPP	2	11	0	2	11	0	0.7	1.3	0.0	0.00	0.00	0.00	0.00	0.00	0.00	UTC	UTC	UTC
EMILIN-1	sp|Q9Y6C2|EMIL1	EMILIN1	24	109	32	24	109	32	7.5	10.9	7.2	18.64	19.04	19.21	0.40	0.57	-0.17	0.280	0.286	0.661

Supplementary Table S4: Gene Ontology analysis results of non-collagen type proteins identified via Collagenase Type III LC-MSMS Proteomics. P-value is Fisher's exact p-value. FDR: false discovery rate

Fold Enrichment	Function	GO	Proteins	Protein Count	p-value	FDR	-LOG10(FDR)
88.65	Basement Membrane	GO:0005604	TNC, LOXL1, HSPG2, BGH3, VTN, NID2, SPARC, FN1, FBN1, FBLN1	10	1.66E-17	1.11E-14	13.95
44.61	Collagen-containing ECM	GO:0062023	TNC, LOXL1, C1QA, TNXB, ASPN, MFAP4, DPT, BGN, FMOD, HSPG2, DCN, LUM, EMILIN1, COMP, BGH3, VTN, NID2, SPARC, FN1, FBN1, FBLN1	21	4.58E-33	9.2E-30	29.04
96.53	Collagen fibril organization	GO:0030199	TNXB, DPT, FMOD, LUM, COMP	5	2.64E-09	5.26E-06	5.28
100	Collagen binding	GO:0005518	ASP, DCN, LUM, COMP, BGH3, VTN, NID2, SPARC, FN1	10	1.24E-16	1.96E-13	12.71
62.06	Collagen metabolism	GO:0010712	MFAP4, VIM, EMILIN-1	3	1.84E-05	0.00666	2.18

Supplemental Table S5: Table outlining which valves were used for each experiment in the manuscript. #: Representative valves in **Supplemental Figure S1**. NI-Not included. Movat's Pentachrome and Herovici stain inclusion were based on sections with sufficient valve structure reporting. Proteomic inclusion was based on system suitability performance. IHC-BAMBI inclusion was done to verify presence of BAMBI protein in representative valve tissue sections. FFPE: Formalin-Fixed Paraffin Embedded. For qPCR studies, see **Supplemental Table S7**.

	SKU	Age (years)	Movat's Pentachrome	Herovici	Picrosirius Red	Proteomics	IHC-BAMBI
**Tissue Type**	-	-	FFPE	FFPE	FFPE	FFPE	FFPE
**Normal**	DB17	0.05	x	NI	x	NI	NI
	DB051	0.66	x	x	x	x	NI
	NDRI	2	x	x		x	x
	DB85	9	x	x	x	x	NI
	DB27	17	x	x	x	x	x
**pCAVS**	DB106	0.04	x	x	x	x	NI
	DB43	0.15	x	x	x	x	NI
	DB40	0.75	x	x	x	x	NI
	DB105	5	x	x	x	x	
	DB119	5	x	x	x	x	x
	DB113	5	#	x	x	x	x
	DB18	6	x	x	x	x	NI
	DB3	11	x	x	x	x	x
	DB117	12	x	x	x	x	NI
	DB41	14	x	x	x	x	x
	DB16	17	x	x	x	x	x
**AVI**	DB114	0.17	x	x	x	x	
	DB007	0.58		x	x	x	
	DB108	10	x	x	x	x	
	DB83	10	x	x	x	x	
	DB032	16	#	x	x	x	

Supplemental Table S6. Primary literature and reviews associated with aortic valve development & disease related to the BAMBI interactive network. Data were acquired by searching Pubmed and Pubmed Central with the gene names AND "aortic valve" or "aortic valve development" of "bicuspid". The majority of the literature on aortic valve disease covers adult calcific aortic valve stenosis and very little has been done on pediatric end-stage aortic valve stenosis.

Gene	Gene Symbol	Role in Aortic Valve	References
Activin receptor-like kinase 2	ACVR1	Depletion in cushion mesenchyme leads to bicuspid aortic valve<br><br>Epithelial-mesenchymal transformation in valve development	[1,2]
Bone Morphogenetic Protein Receptor Type 1A	BMPR1A	Activation required for aortic valve calcification	[3]
B-catenin	CTNNB1	Endocardial cushion development<br><br>Pressure overload induced fibrosis	[4,5]
Dishevelled Segment Polarity Protein 2	DVL2	Upregulated in adult stenotic aortic valves	[6]
Estrogen Receptor Beta	ESR2	Increases in patients with aortic valve stenosis<br><br>Sex specific disease features in adult aortic valve disease	[7] ;Review[8]
Histone Acetylation and Deacetylation	HDAC1	Epigenomic alterations in aortic valve stenosis	[9]
LDL Receptor Related Protein 6	LRP6	Essential for outflow tract development<br><br>Increased receptor expression in adult calcific aortic valve<br><br>Promotes calcification in aortic valve	[10,11] ; Review[12]
Lamin A/C	LMNA	Genetic mutation with congenital aortic valve malformation<br><br>Activation of osteogenic differentiation in aortic valve interstitial cells<br><br>Decreased in bicuspid aortic valve	[13-15]
Nuclear Factor kappa B	NFκB	Valvular endocardial cushion development<br><br>Cell type differentiation during calcific aortic valve disease<br><br>Inflammatory regulation in early calcific aortic valve disease	[16-19]
SMAD Family Member 6	SMAD6	Epithelial-mesenchymal transformation in valve development<br><br>Alteration of ECM in aortic development<br><br>Variants in congenital aortic valve disease	[2,20-22]
SMAD Family Member 7	SMAD7	Outflow tract malformations<br><br>Increases valvular calcification with the downregulation of miR-195 by SMAD7 targeting	[23,24]
SWI/SNF Related, Matrix Associated, Actin Dependent Regulator of Chromatin, Subfamily A, Member 4	SMARCA4	Mutational effects in bicuspid aortic valve	[25]
Transforming Growth Factor Beta Receptor 2	TGFBR2	Epithelial-mesenchymal transformation in valve development & disease<br><br>Ligand regulation of AV cushion endocardial cells<br><br>Mutations in bicuspid aortic valve	[26-28]

REFERENCES

1 Thomas, P. S., Sridurongrit, S., Ruiz-Lozano, P. & Kaartinen, V. Deficient signaling via Alk2 (Acvr1) leads to bicuspid aortic valve development. *PloS One* **7**, e35539 (2012).

2 Desgrosellier, J. S., Mundell, N. A., McDonnell, M. A., Moses, H. L. & Barnett, J. V. Activin receptor-like kinase 2 and Smad6 regulate epithelial–mesenchymal transformation during cardiac valve formation. *Developmental Biology* **280**, 201-210 (2005).

3 Gomez-Stallons, M. V., Wirrig-Schwendeman, E. E., Hassel, K. R., Conway, S. J. & Yutzey, K. E. Bone morphogenetic protein signaling is required for aortic valve calcification. *Arteriosclerosis, Thrombosis, and Vascular Biology* **36**, 1398-1405 (2016).

4 Liebner, S. *et al.* β-catenin is required for endothelial-mesenchymal transformation during heart cushion development in the mouse. *Journal of Cell Biology* **166**, 359-367 (2004).

5 Xiang, F.-L., Fang, M. & Yutzey, K. E. Loss of β-catenin in resident cardiac fibroblasts attenuates fibrosis induced by pressure overload in mice. *Nature Communications* **8**, 1-12 (2017).

6 Khan, K. *et al.* The role of Wnt/β-catenin pathway mediators in aortic valve stenosis. *Frontiers in Cell and Developmental Biology* **8**, 862 (2020).

7 Nordmeyer, J. *et al.* Upregulation of myocardial estrogen receptors in human aortic stenosis. *Circulation* **110**, 3270-3275 (2004).

8 Summerhill, V. I., Moschetta, D., Orekhov, A. N., Poggio, P. & Myasoedova, V. A. Sex-specific features of calcific aortic valve disease. *International Journal of Molecular Sciences* **21**, 5620 (2020).

9 Gošev, I. *et al.* Epigenome alterations in aortic valve stenosis and its related left ventricular hypertrophy. *Clinical Epigenetics* **9**, 1-28 (2017).

10 Rajamannan, N. M. The role of Lrp5/6 in cardiac valve disease: experimental hypercholesterolemia in the ApoE−/−/Lrp5−/− mice. *Journal of Cellular Biochemistry* **112**, 2987-2991 (2011).

11 Song, L., Li, Y., Wang, K. & Zhou, C. J. Cardiac neural crest and outflow tract defects in Lrp6 mutant mice. *Developmental Dynamics* **239**, 200-210 (2010).

12 Kang, S. Low-density lipoprotein receptor-related protein 6-mediated signaling pathways and associated cardiovascular diseases: diagnostic and therapeutic opportunities. *Human Genetics* **139**, 447-459 (2020).

13 Tao, J., Duan, J., Pi, X., Wang, H. & Li, S. A splicing LMNA mutation causing laminopathies accompanied by aortic valve malformation. *Journal of Clinical Laboratory Analysis*, e23736 (2021).

14 Perepelina, K., Klauzen, P., Kostareva, A. & Malashicheva, A. Tissue-specific influence of lamin a mutations on notch signaling and osteogenic phenotype of primary human mesenchymal cells. *Cells* **8**, 266 (2019).

15 Grewal, N. *et al.* Ascending aorta dilation in association with bicuspid aortic valve: a maturation defect of the aortic wall. *The Journal of Thoracic and Cardiovascular Surgery* **148**, 1583-1590 (2014).

16 DeLaughter, D. M. *et al.* Spatial transcriptional profile of the chick and mouse endocardial cushions identify novel regulators of endocardial EMT in vitro. *Journal of Molecular and Cellular Cardiology* **59**, 196-204 (2013).

17 Mahler, G. J., Farrar, E. J. & Butcher, J. T. Inflammatory cytokines promote mesenchymal transformation in embryonic and adult valve endothelial cells. *Arteriosclerosis, Thrombosis, and Vascular Biology* **33**, 121-130 (2013).

18 Gee, T. *et al.* Nfκb (nuclear factor κ-light-chain enhancer of activated b cells) activity regulates cell-type–specific and context-specific susceptibility to calcification in the aortic valve. *Arteriosclerosis, Thrombosis, and Vascular Biology* **40**, 638-655 (2020).

19 Kaden, J. J. *et al.* Receptor activator of nuclear factor κB ligand and osteoprotegerin regulate aortic valve calcification. *Journal of Molecular and Cellular Cardiology* **36**, 57-66 (2004).

20 Sugi, Y., Kern, M. J., Markwald, R. R. & Burnside, J. L. Periostin expression is altered in aortic valves in Smad6 mutant mice. *Journal of Neonatal Biology* **1** (2012).

21 Sen, D. G. *et al.* The transcriptional signature of growth in human fetal aortic valve development. *The Annals of Thoracic Surgery* **106**, 1834-1840 (2018).

22 Park, J. E. *et al.* A novel SMAD6 variant in a patient with severely calcified bicuspid aortic valve and thoracic aortic aneurysm. *Molecular Genetics & Genomic Medicine* **7**, e620 (2019).

23 Chen, Q. *et al.* Smad7 is required for the development and function of the heart. *J Biol Chem* **284**, 292-300 (2009).

24 Du, J. *et al.* Downregulated MicroRNA-195 in the bicuspid aortic valve promotes calcification of valve interstitial cells via targeting SMAD7. *Cellular Physiology and Biochemistry* **44**, 884-896 (2017).

25 Preuss, C. *et al.* Family based whole exome sequencing reveals the multifaceted role of notch signaling in congenital heart disease. *PLoS Genetics* **12**, e1006335 (2016).

26 Sanford, L. P. *et al.* TGFbeta2 knockout mice have multiple developmental defects that are non-overlapping with other TGFbeta knockout phenotypes. *Development* **124**, 2659-2670 (1997).

27 Brown, C. B., Boyer, A. S., Runyan, R. B. & Barnett, J. V. Antibodies to the type II TGFβ receptor block cell activation and migration during atrioventricular cushion transformation in the heart. *Developmental Biology* **174**, 248-257 (1996).

28 Girdauskas, E., Schulz, S., Borger, M. A., Mierzwa, M. & Kuntze, T. Transforming growth factor-beta receptor type II mutation in a patient with bicuspid aortic valve disease and intraoperative aortic dissection. *The Annals of Thoracic Surgery* **91**, e70-e71 (2011).

Supplemental Table S7: Descriptive statistics of patient characteristics for aortic valve samples used for rt-qPCR studies (**Fig. 6**). Normal aortic valves are from cardiac transplant patients where normal valve function is defined via 2D echocardiogram. 2D Echocardiogram mean gradients define pre-operative valve function where a normal gradient is defined as <5 mmHg, mild stenosis as 5-25 mmHg, moderate stenosis as 25-50 mmHg, and severe stenosis as >50 mmHg. BSA: body surface area; C: Caucasian. DB43 was included in pCAVS due to stenosis, but main diagnosis was Hypertrophic Cardiomyopathy

	SKU	Age (years)	Pediatric Age Group	Gender	Race/ Ethnicity	BSA (m^2)	Leaflet Morphology	Aortic Valve Function
Normal	DB85	9	Child	F	C	0.95	Trileaflet	Normal
	DB27	17	Adolescent	M	C	2.2	Trileaflet	Normal
	**Normal Averages**	**7.17 ± 7.5**		**75%M 25%F**	**75%C 25%L**	**1.03**		
pCAVS	DB43	0.15	Infant	M	C	0.24	Trileaflet	Moderate stenosis with AVI*
	DB40	0.75	Infant	M	C	0.37	Bicuspid	Moderate Stenosis
	DB105	5	Young Child	M	C	0.72	Bicuspid	Moderate Stenosis with AVI
	DB119	5	Young Child	F	-	-	Bicuspid	Moderate Stenosis with AVI
	DB113	5	Young Child	-	-	-	-	Trivial Stenosis; Severe AVI
	DB18	6	Child	F	C	0.7	Bicuspid	Moderate Stenosis with AVI
	DB117	12	Adolescent	M	-		Bicuspid	Moderate Stenosis with AVI
	DB41	14	Adolescent	M	C	1.6	Bicuspid	Moderate Stenosis with AVI
	DB135	17	Adolescent	M	-	-	-	Moderate Stenosis with AVI
	DB136	17	Adolescent	M	-	-	Bicuspid	Moderate Stenosis with AVI
	DB162	17	Adolescent	F	-	-	Bicuspid	Moderate Stenosis with AVI
	DB191	17	Adolescent	M	-	-	Bicuspid	Moderate Stenosis with AVI
	**pCAVS Averages**	**6.90 ± 5.8**		**73%M 27%F**	**100%C**	**0.93**		
aCAVS	DB226	19	Adult	F	-	-	Trileaflet	Moderate Stenosis; no calcification
	DB176	25	Adult	F	-	-	Trileaflet	Moderate Stenosis with AVI; calcification
	DB174	26	Adult	M	-	-	Bicuspid	Moderate Stenosis; no calcification
	DB221	26	Adult	M	-	-	Bicuspid	Moderate Stenosis with AVI; no calcification
	DB284	36	Adult	F	-	-	Bicuspid	Severe Stenosis; no calcification
	**aCAVS Averages**	**26.4 ± 6.1**		**40%M 60%F**	**100%C**	**-**		
**Ross**	DB152	22	Adult	M	-	-		Pulmonary-AV Autograft with AVI
